# Far-red perception by vegetative organs and not fruits drives fruit growth responses in tomato plants

**DOI:** 10.1093/plphys/kiag358

**Published:** 2026-06-15

**Authors:** Elena Vincenzi, Lisa Oskam, Mohan Lu, Ronald Pierik, Esther de Beer, Frank Millenaar, Leo F M Marcelis, Ep Heuvelink

**Affiliations:** Horticulture and Product Physiology, Wageningen University and Research, Wageningen, Netherlands; Laboratory of Molecular Biology, Wageningen University and Research, Wageningen, Netherlands; Horticulture and Product Physiology, Wageningen University and Research, Wageningen, Netherlands; Laboratory of Molecular Biology, Wageningen University and Research, Wageningen, Netherlands; Signify, Plant Specialist Team, Eindhoven, Netherlands; BASF—Nunhems, Pre-Breeding Team, Nunhem, Netherlands; Horticulture and Product Physiology, Wageningen University and Research, Wageningen, Netherlands; Horticulture and Product Physiology, Wageningen University and Research, Wageningen, Netherlands

## Abstract

Responses to far-red light are mediated by phytochromes, specialized photoreceptors present in all organs of a tomato plant (*Solanum lycopersicum* L.). Although fruit-localized phytochromes can influence starch and sugar metabolism, their involvement in modulating fruit growth responses to far-red light is unknown. We explored whether fruit growth responses to far-red light are driven by local far-red perception within the fruits or by a systemic response initiated in the vegetative organs. We applied far-red light exclusively to the fruiting trusses, to the vegetative organs, to both, or to neither. Far-red light was added to a constant red–white LED background spectrum to simulate greenhouse supplementary lighting. We quantified plant and fruit growth responses to far-red and performed a transcriptomic analysis to establish the temporal dynamics of far-red light perception and signaling in leaves and fruits. Supplementing far-red light to the vegetative organs increased ripe fruit weight and sugar concentration, whereas far-red supplementation to the generative organs alone had no measurable effect. This study establishes that far-red perception by vegetative organs drives fruit growth responses in tomato. Local responses to far-red light included a transient increase in auxin concentration and upregulation of the auxin-signaling pathway in leaves, a conserved response to decreasing red/far-red ratios. In fruits, early transcriptomic responses carried signatures of auxin, cytokinin, and gibberellin biosynthesis and signaling, suggesting a role for hormones as mediators of far-red-induced fruit responses. This work reveals how responses to far-red light perception by vegetative organs promote sink strength and starch biosynthesis in early developing fruits, contributing to higher fruit weight and sugar concentration in ripe fruits.

## Teaser

Vegetative organs are the key sites perceiving far-red light enrichment, which triggers subsequent fruit growth responses in tomato plants.

## Introduction

Unfiltered solar light presents almost equal proportions of red (R, 600 to 700 nm) and far-red light (FR, 700 to 800 nm), and their ratio is perceived in plants by a class of photoreceptors called phytochromes (Casal 2000). Phytochromes can exist in two stable but photo-interconvertible forms: a biologically inactive form (Pr) with peak absorption in the red spectrum and a biologically active form (Pfr) with peak absorption in the FR spectrum (Rockwell et al. 2006). Phytochromes in their active state translocate from the cytosol to the cell nucleus, where they interact with a wide light-signaling regulatory network, ultimately influencing plant development, from seed germination to senescence (Weller et al. 2000; Rosado et al. 2016).

In natural settings, the decrease in the R to FR ratio (R:FR) of perceived light provides crucial environmental cues, informing plants of potential competition or shading and triggering adaptive strategies to increase their chances of survival (Casal and Fankhauser 2023). These include mostly stem and petiole elongation responses, as well as reduced branching, and are collectively referenced to as shade-avoidance responses (Ballaré and Pierik 2017; Fernández-Milmanda and Ballaré 2021). Conversely, in controlled-environment agriculture, the light spectrum experienced by plants is traditionally rich in red light while lacking ultraviolet and FR. When FR is added to red-rich LED lighting regimes (FR enrichment), the R:FR perceived by the plants decreases while typically remaining well above that of direct solar light (R:FR ∼ 1.2). Far-red enrichment can elicit morphological and developmental responses, offering the potential to improve yield in multiple crops, including tomato (Demotes-Mainard et al. 2016; Zhang et al. 2024). Despite differing from shade conditions, where R:FR decreases below that of solar light, responses to FR enrichment are widely attributed to phytochrome signaling. However, it remains unclear to what extent plant responses are comparable across these different ranges of R:FR ratios.

Tomato (*Solanum lycopersicum* L.) is one of the most widely consumed and economically valuable crops worldwide and is considered a model species for fleshy fruit-bearing plants. Under FR enrichment, several studies have reported increases in tomato fruit yield, primarily attributed to a shift in dry matter partitioning toward the fruits (Ji et al. 2019) and in some cases also to enhanced plant dry weight production (Kim et al. 2019; Vincenzi et al. 2024, 2025). The latter process is largely driven by increased light use efficiency, combining physiological and morphological components, such as a higher leaf CO_2_ assimilation rate due to the enhancement of photosynthesis under FR enrichment and higher light interception due to changes in plant architecture (Emerson and Rabinowitch 1960; Kalaitzoglou et al. 2019; Zhen et al. 2022). The shift in dry matter partitioning has been linked to an increase in fruit sink strength, defined as the competitive ability of an organ to attract assimilates from the plant assimilate pool (Marcelis 1996; Ji et al. 2020). These responses collectively result in greater individual fruit weight and increased sugar concentration in ripe fruits under FR-enriched conditions (Fanwoua et al. 2019; Ji et al. 2020).

Plant responses to FR enrichment may be initiated by local FR perception or by the systemic transmission of a molecular signal from the light-perceiving organ to the responding organ (Küpers et al. 2018). Petiole elongation exemplifies a local FR response, where both the perception of the light signal and the regulatory response occur in the same location (Pantazopoulou et al. 2017). In contrast, petiole hyponasty, hypocotyl elongation, accelerated flowering transition, and reduced lateral root growth require signal communication across different tissues or organs, reaching even opposite sides of a plant (Tanaka et al. 2002; Endo et al. 2005; Procko et al. 2014; van Gelderen et al. 2018; Küpers et al. 2023; van der Meer et al. 2023; Gautrat et al. 2024).

The tomato genome harbors five *PHYTOCHROME* (*SlPHY*) genes, *SlPHYA*, *SlPHYB1*, *SlPHYB2*, *SlPHYE*, and *SlPHYF*, which are expressed at varying levels across all plant organs (Pratt et al. 1997). Functional studies at the whole-plant level, involving the silencing of one or more phytochromes, have revealed their complex role in controlling fruit carotenoid and chlorophyll accumulation and the length of the ripening period (Gupta et al. 2014; Bianchetti et al. 2017). To further explore organ-specific roles, fruit-specific phytochrome mutants and detached fruit systems have been used to distinguish local from systemic phytochrome contributions. These studies have demonstrated a role for fruit-localized phytochromes in sugar metabolism and plastid biogenesis during early fruit development and carotenoid and lycopene biosynthesis during ripening (Alba et al. 2000; Ernesto Bianchetti et al. 2018; Alves et al. 2020). Despite the increasing body of knowledge, it remains unknown whether FR enrichment promotes fruit yield and alters dry matter partitioning in tomato plants through a fruit-localized or systemic response (Heuvelink et al. 2025).

In this study, we aimed to investigate whether tomato fruit growth responses to FR enrichment are mediated by fruit-localized FR perception or if they result from interorgan communication of a FR signal perceived in a different organ of the plant. We hypothesized that FR perception by vegetative organs would be required to promote plant dry weight production, while FR perception by generative organs (flowering and fruiting trusses) would increase the dry matter partitioning to the fruits by enhancing fruit sink strength, ultimately leading to higher fruit sugar content. To determine the location of FR perception responsible for mediating fruit growth responses, we selectively exposed generative and vegetative organs of fruiting tomato plants to supplemental FR and studied the associated physiological and transcriptomic responses. Our data showed that FR perception by vegetative organs was necessary to mediate plant and fruit growth responses, while localized FR application on tomato trusses, from anthesis to ripe fruits, showed no significant effect. Far-red perception by vegetative organs increased sucrose import and starch accumulation in early developing fruits and was associated with upregulation of sink and starch biosynthetic genes. Consequently, FR enrichment promoted individual fruit weight and sugar content in ripe fruits. A time–course transcriptomic experiment further elucidated the dynamics of interorgan FR signaling from the vegetative to the generative organs, providing evidence for hormonal regulation as part of the early fruit response.

## Results

### Far-red light perception by vegetative organs drives fruit growth responses

**Figure 1:**
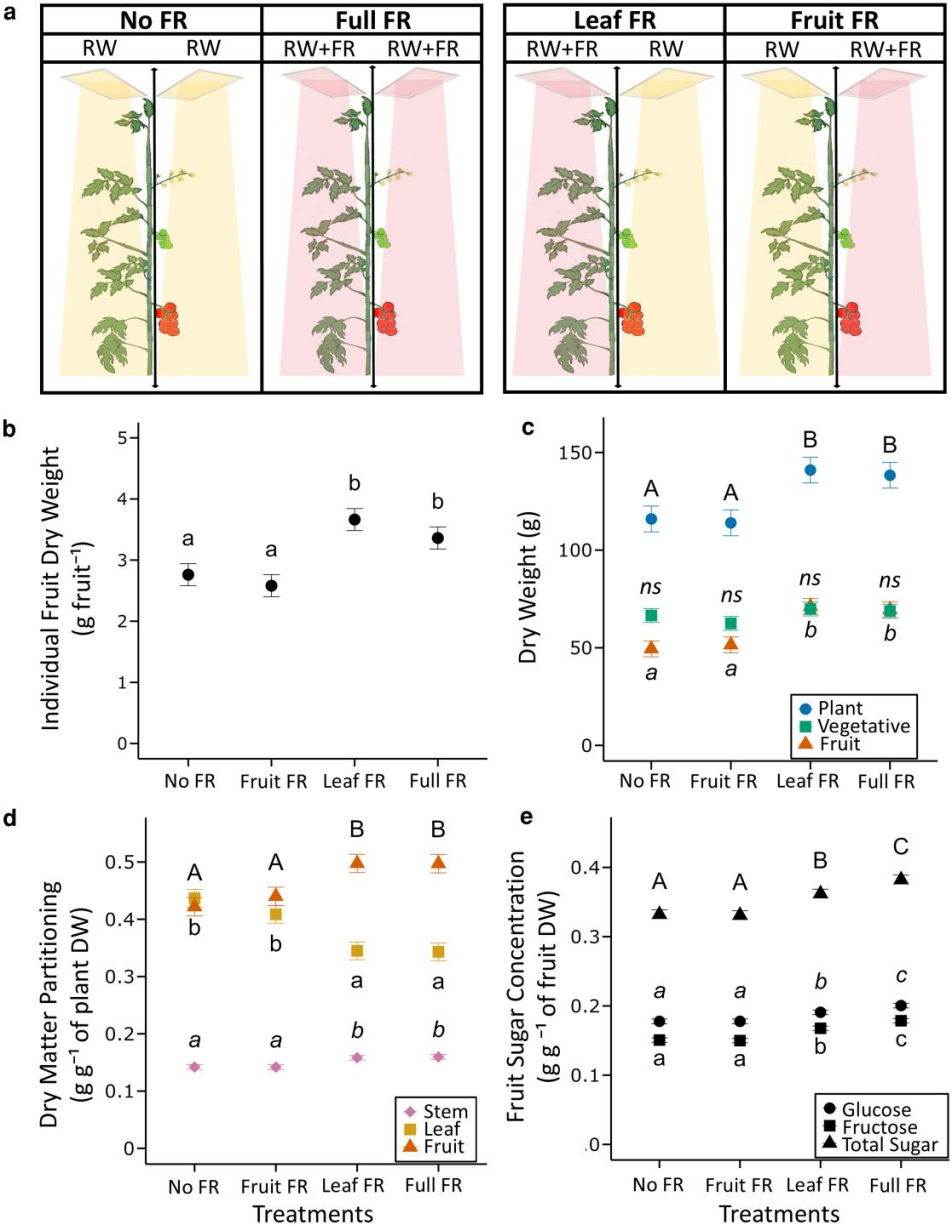
Experimental setup and effects of far-red light (FR) treatments on dry weight production and partitioning between the plant organs and fruit sugar concentration. (a) Experimental setup, FR was applied together with red and white (RW) light on the vegetative organs of the plants (Leaf FR), on the generative organs of the plants (Fruit FR), on both of them (Full FR), or on neither of them (No FR). (b) Individual ripe fruit dry weight. (c) Dry weight of aerial organs (fruit—▴, vegetative—■, plant—●). (d) Dry matter partitioning to the aerial organs (fruit—▴, leaf—■, stem—◆). (e) Ripe fruit sugar concentration (total—▴, fructose—■, glucose—●). Values of fruit dry weight and fruit sugar concentration are averages of two trusses per plant. Different letters indicate significant differences according to Fisher's protected LSD test (*P* = 0.05). Labels “*ns*” indicate no significant differences among the treatments. Values represent averages of three to four experimental units ± SEM (n = 3 for Fruit FR and Leaf FR, n = 4 for No FR and Full FR), with each experimental unit consisting of four plants.

We investigated the role of vegetative and generative organs of tomato plants in mediating fruit growth responses to supplementary FR light. To do so, we developed an experimental setup that allowed us to separate the vegetative and generative organs of the same tomato plant by a light-impermeable curtain and to expose them to different light conditions. We tested four light treatments, with FR being added to a red and white (RW) background light on only the vegetative organs (Leaf FR), only the generative organs (Fruit FR), both (Full FR), or neither of them (No FR) (Fig. 1a, Figure S1). Compared to the No FR treatment, FR supplementation reduced the R:FR ratio perceived by plants while remaining well above solar R:FR (Table 1). All plants received only RW light until the anthesis of the first flower of the second truss, when the FR treatment began. Fruits from the first and second truss were individually harvested at the light-red ripe stage (USDA, 1991) and analyzed together since they showed similar trends. Application of FR to the vegetative organs led to an increase in individual fruit fresh and dry weight compared to No FR, regardless of whether the fruits themselves were exposed to FR (Fig. 1b, Figure S2a). Against our hypothesis, exposing only the fruiting trusses to supplementary FR did not significantly affect fruit weight compared to the No FR treatment. The increase in fruit weight observed under both Leaf FR and Full FR treatments was the main reason for the increase in plant dry weight, while the dry weight of vegetative organs (leaves, stem, and apex) remained unchanged (Fig. 1c). Consequently, the proportion of dry weight partitioned to the fruits increased, at the expense of the leaves (Fig. 1d). The increase in plant dry weight in Leaf FR and Full FR treatments is in line with the higher leaf CO_2_ assimilation rate recorded under FR enrichment (Figure S3).

**Table 1:** Photosynthetic photon flux density (PPFD; 401 to 700 nm), photon flux density (PFD) of far-red (PFD-FR; 701 to 800 nm), FR fraction, R:FR ratio, and phytochrome photostationary state (PSS; Sager et al. 1988) of the light treatments used in this research. Light treatments involved specific settings for the vegetative (Leaves = leaves, stem, and apex) and generative organs (Fruits = flowering and fruiting trusses) of each plant. Values represent average ± SEM (Expt. 1: n = 3 units for Fruit FR and Leaf FR, n = 4 units for No FR and Full FR; Expt. 2: n = 6 units; each unit consisted of four plants). SEMs < 0.001 were omitted from the table.

Expt.	Light treatments		PPFD	PFD-FR	FR fraction	R:FR	PSS
			(μmol m^−2^ s^−1^)	(μmol m^−2^ s^−1^)	FR:(R + FR)		
**1**	No FR	Leaves Fruits	222 ± 5 222 ± 5	1.7 ± 0.2 1.7 ± 0.2	0.01 0.01	116 ± 14 116 ± 14	0.88 0.88
	Fruit FR	Leaves Fruits	202^a^ ± 12 222 ± 8	1.4 ± 0.1 82 ± 1	0.01 0.31 ± 0.001	121 ± 12 2.3 ± 0.01	0.88 0.79
	Leaf FR	Leaves Fruits	222 ± 8 202^a^ ± 12	82 ± 1 1.4 ± 0.1	0.31 ± 0.001 0.01	2.3 ± 0.01 121 ± 12	0.79 0.88
	Full FR	Leaves Fruits	215 ± 8 215 ± 8	76 ± 276 ± 2	0.30 ± 0.001 0.30 ± 0.001	2.5 ± 0.02 2.5 ± 0.02	0.79 0.79
**2**	No FR	Leaves Fruits	260 ± 2 260 ± 2	0.7 ± 0.1 0.7 ± 0.1	0.004 0.004	383 ± 30 383 ± 30	0.88 0.88
						383	
	Leaf FR	Leaves Fruits	257 ± 2 260 ± 2	77 ± 1 0.7 ± 0.1	0.28 ± 0.001 0.004	2.6 ± 0.01 383 ± 30	0.80 0.88

^a^PPFD was lower than average in one unit due to ceiling lighting constraints. Differences in PPFD due to the experimental setup were addressed by including PPFD as a covariate in the statistical analysis.

**Figure 2:**
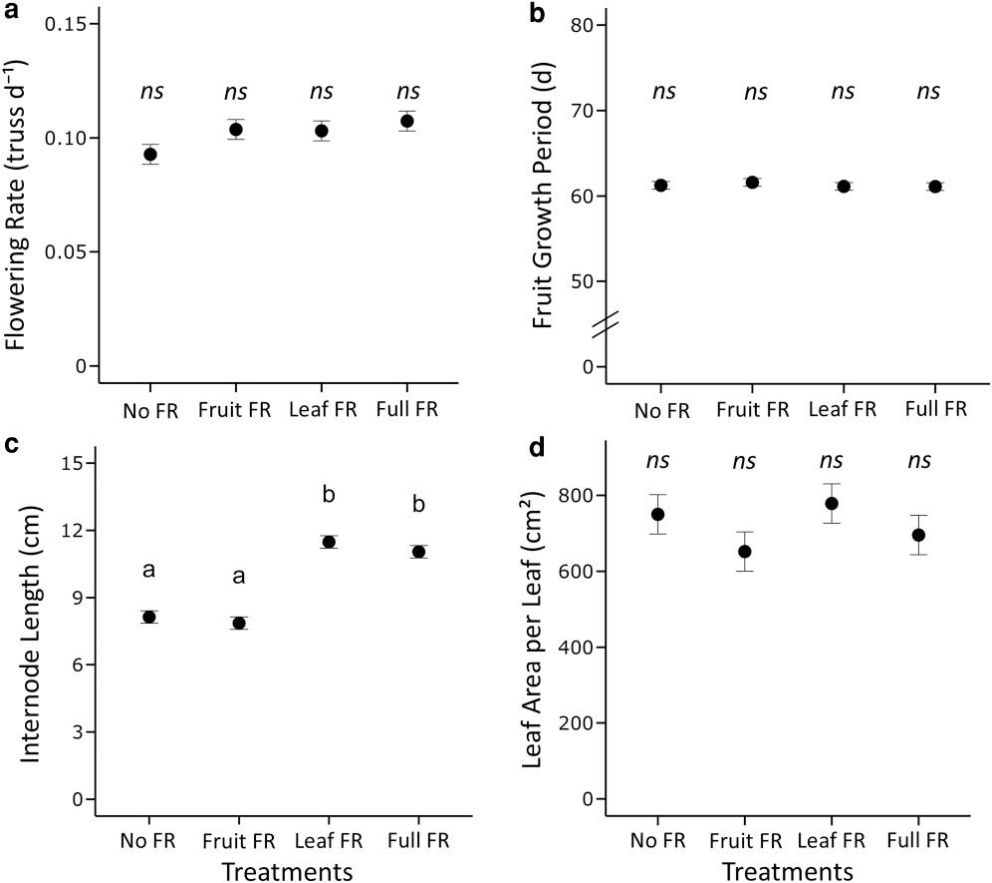
Effects of far-red light (FR) treatments on plant architecture and development. (a) Flowering rate. (b) Fruit growth period. (c) Internode length, representing the average internode length measured between node 15 and node 25, at the end of the experiment (fully developed internodes). (d) Leaf area per leaf. Different letters indicate significant differences according to Fisher's protected LSD test (*P* = 0.05). Labels “*ns*” indicate no significant differences among the treatments. Values represent averages of three to four experimental units ± SEM (n = 3 for Fruit FR and Leaf FR, n = 4 for No FR and Full FR), with each experimental unit consisting of four plants.

Fruit sugar concentration at harvest was significantly higher when FR was applied to the vegetative organs (Fig. 1e), while fruit dry matter content was not affected (Figure S2b). In the No FR treatment, fruit sugar content consisted of approximately 45% fructose, 54% glucose, and only 1% sucrose, with starch levels below the detection threshold. Far-red application to vegetative organs increased both fructose and glucose concentrations on a dry weight basis, though not equally, resulting in a higher glucose-to-fructose ratio in ripe fruits under the Leaf FR and Full FR treatments as compared to No FR (Figure S2c). In contrast, FR application to generative organs had no significant effect on fruit sugar concentration or composition. Notably, the Full FR treatment had the highest total sugar concentration, significantly exceeding that observed under Leaf FR alone (Fig. 1e). Neither flowering rate nor the duration of fruit growth (from anthesis to harvest) was significantly affected by any FR treatment (Fig. 2a and b). On the other hand, FR application to the vegetative organs significantly increased internode length (Fig. 2c) and the proportion of plant dry weight partitioned to the stem (Fig. 1d) while having no impact on leaf area per leaf (Fig. 2d).

Taken together, our results show that FR supplementation increases individual ripe fruit weight and sugar concentration, but only when the vegetative organs are exposed to FR. This effect is associated with an increase in both plant dry weight and dry matter partitioning to the fruits. While the former process reflects an increase in assimilate production (source strength), the latter suggests an increase in fruit sink strength, especially considering that fruit number was kept consistent across all treatments. Therefore, these results indicate that fruit growth response to FR enrichment is a systemic process that integrates the perception of FR in the vegetative organs and the communication of this perception to the fruits, where it promotes fruit sink strength.

### Leaf perception of far-red light leads to delayed gene expression regulation in fruits

**Figure 3:**
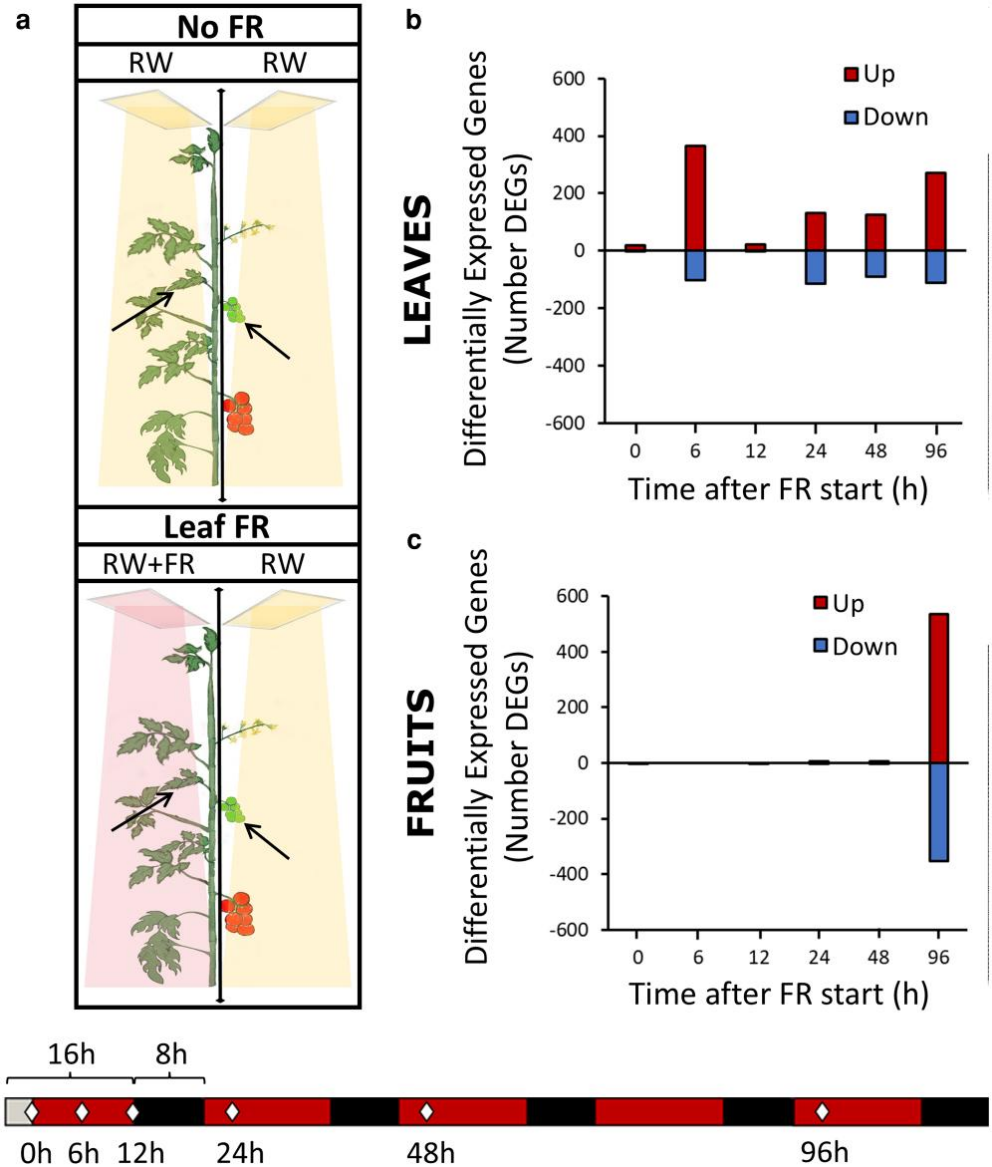
Leaf and fruit transcriptomic responses to far-red (FR) light applied to vegetative organs (Leaf FR). (a) Plants under Leaf FR or No FR were grown under the same red and white (RW) light until the start of the FR treatment, 63 days after sowing. FR started 4 h after the beginning of the RW photoperiod (16/8 day/night) and thereafter followed the same timing. Leaf and fruit samples were collected at six time points from pre-exposure (0) to 96 h after the start of FR treatment. (b) Differentially expressed genes (DEGs) identified in leaves. (c) Differentially expressed genes (DEGs) identified in fruits. DEGs were identified for each organ and time point by comparing Leaf FR vs. NoFR, using an adjusted *P*-value threshold of 0.05.

To investigate interorgan communication of FR perception between vegetative and generative organs of a tomato plant and its role in controlling fruit growth responses, we studied the associated transcriptomic response. We analyzed changes in gene expression in leaves and fruits under two of the four light treatments: Leaf FR and No FR. Leaf and fruit samples were collected at six time points: 0 (pretreatment), 6, 12, 24, 48, and 96 hours after the start of the FR treatment (Fig. 3a, Supplementary Dataset S1 to S3). Differentially expressed genes (DEGs) were identified separately for each organ and time point by comparing Leaf FR to No FR, using an adjusted *P*-value threshold of 0.05 (Supplementary Dataset S4). In the leaves, the number of DEGs peaked at the earliest time point (6 h), dropped sharply at 12 h, and then rose again, remaining relatively stable at all successive time points (Fig. 3b). This pattern may reflect an interaction between the response to FR and the diurnal regulation of gene expression, as sampling at 0, 24, 48, and 96 h occurred 4 h after the start of the photoperiod, while the 12-h sampling was conducted just before the end of the photoperiod. The temporal trend we observed in leaf DEGs held true regardless of the expression threshold applied, considering only highly DEGs (Log_2_FC > 1 or < −1; Figure S4) or all DEGs (Fig. 3b). In contrast, the fruit transcriptome showed a clear delay in response to leaf perception of FR, with only 15 genes differentially expressed prior to the last time point (Fig. 3c). At 96 h, the number of DEGs increased to more than 800 (535 upregulated and 353 downregulated). Interestingly, most fruit DEGs, in particular the upregulated ones, had a Log_2_FC included between −1 and 1, and only from 96 h. This suggests that transcriptional changes in the fruits were only just beginning at 96 h after the start of Leaf FR treatment, indicating a delayed regulatory response. Altogether, direct exposure to FR resulted in strong and persistent gene expression regulation in leaves, with the highest number of DEGs recorded at the first time point. In contrast, fruits, which were not exposed to FR, exhibited transcriptomic changes only after a delay of more than 48 h, suggesting a relatively slow interorgan communication in which FR perception in leaves triggers downstream events that eventually modulate gene expression in fruits.

### Far-red perception transiently increases free auxin levels and activates auxin-responsive pathways in leaves

**Figure 4:**
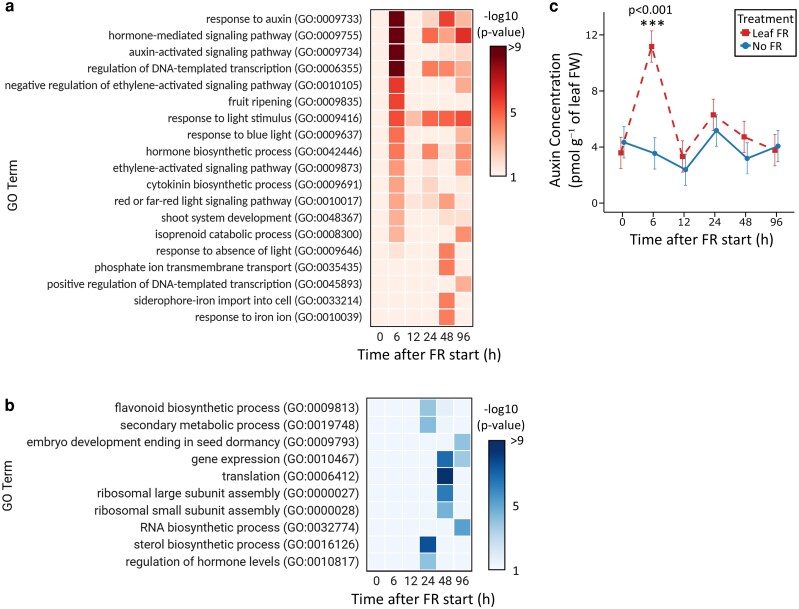
Leaf response to direct illumination by far-red light (Leaf FR treatment), per time point. (a) Gene Ontology (GO) enrichment analysis of genes upregulated by the FR treatment. (b) GO enrichment analysis of genes downregulated by the FR treatment. The displayed GO categories were significantly enriched (adjusted *P*-value threshold of 0.05) among the up- or downregulated genes at least for one time point. (c) Auxin (indole-3-acetic acid) concentration in leaf samples. Auxin concentration values were analyzed with a two-way analysis of variance (ANOVA), and asterisks represent a significant difference according to Fisher's protected LSD test (*P* = 0.05). Values represent the average of four replicates ± SEM (n = 4).

To investigate leaf molecular responses potentially involved in interorgan communication of FR perception, we conducted a Gene Ontology (GO) term enrichment analysis on genes that were significantly up- or downregulated by Leaf FR at each time point (Fig. 4a, b, Supplementary Dataset S5). Among the upregulated genes, several were annotated with hormone-related GO terms, consistent with the established role of hormones in mediating light signaling in plants (Fernández-Milmanda and Ballaré 2021). In particular, GO terms related to auxin response and auxin-activated signaling pathway were enriched at three to four time points, with the strongest enrichment occurring at the earliest time point (Fig. 4a). In addition to auxin-related terms, categories associated with ethylene signaling and cytokinin biosynthetic process were also significantly enriched. Among the other enriched categories, several were related to light (“response to light stimulus,” “response to blue light,” “red or far-red light-signaling pathway,” and “response to absence of light”) and regulation of transcription. Early upregulation of auxin-responsive genes aligns with known shade-avoidance mechanisms in *Arabidopsis* (de Wit et al. 2015; Kohnen et al. 2016; Iglesias et al. 2018), supporting a conserved role for auxin as a key mediator of FR signaling. To confirm auxin involvement in the rapid response to FR perception, we quantified the level of free indole-3-acetic acid (IAA) in the same leaf samples used for transcriptomic analysis. We observed a significant increase in free IAA concentration at 6 h after the start of the Leaf FR treatment (Fig. 4c). However, at 12 h, IAA levels were no longer significantly different from those in the No FR control. A fast and transient rise in IAA levels in response to low R:FR in vegetative organs is consistent with previous shade-avoidance work in *Arabidopsis* (Bou-Torrent et al. 2014; Pucciariello et al. 2018). There were no GO terms significantly enriched (adjusted *P*-value threshold = 0.05) among the downregulated genes under Leaf FR treatment at the early time points, with the first ones being at 24 h after FR start (Fig. 4b). Enriched GO terms among the downregulated genes were related to gene expression and protein synthesis, as well as hormonal regulation and the biosynthesis of secondary metabolites such as flavonoids and sterols.

**Figure 5:**
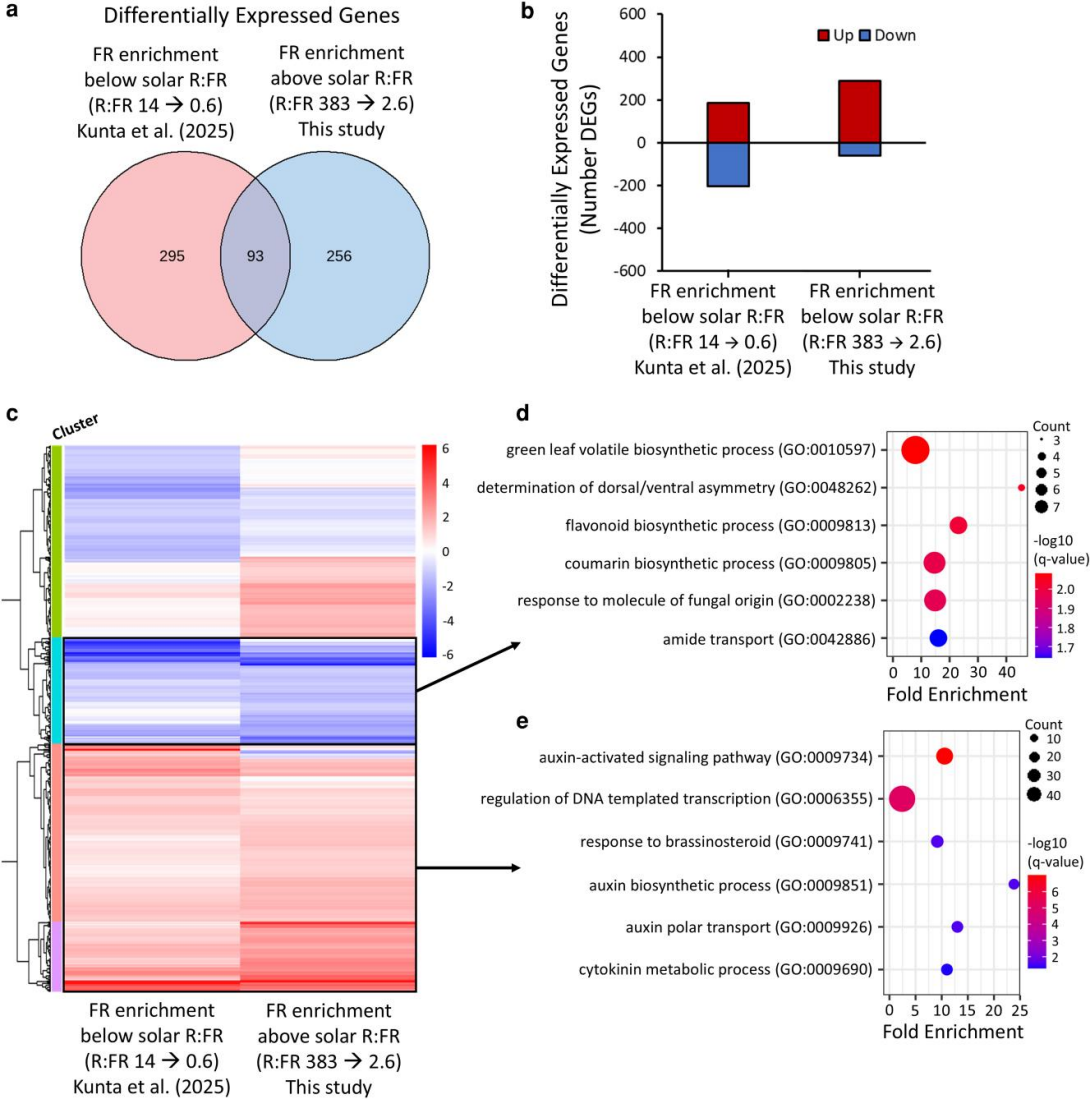
Comparison of leaf transcriptomic responses 6 h after starting far-red (FR) light enrichment (white light + FR vs. white light) below solar R:FR (Kunta et al. 2025) or above solar R:FR (this study). Genes highly differentially expressed (Log_2_FC > 1 or < −1, adjusted *P*-value threshold of 0.05) in Kunta et al. (2025) and/or this study, but present in both, were selected to illustrate expression patterns. (a) Venn diagram, (b) bar plot, and (c) heatmap representation of these selected genes. In total, 61 genes were expressed in only one of the studies; these genes were not plotted in the heatmap but were included in Supplementary Dataset S6. (d) Gene Ontology (GO) categories significantly enriched (adjusted *P*-value threshold of 0.05) among gene cluster showing similar downregulation between the two studies. (e) GO categories significantly enriched among gene clusters showing similar upregulation between the two studies.

To contextualize these findings, we compared our peak transcriptomic response (6 h) with a study by Kunta et al. (2025) (Fig. 5). While both studies applied FR enrichment to lower the R:FR ratio, the resulting light environments represented distinct physiological windows. Specifically, our treatment maintained R:FR values consistently above that of direct solar light (∼ 1.2), decreasing from a R:FR of 383 to 2.6, upon FR enrichment. In contrast, Kunta et al. (2025) reached a R:FR ratio below solar R:FR, decreasing from 14 to 0.6. A similar number of genes were highly differentially expressed in each study, and about one quarter of these genes were common between the two (Fig. 5a). Under FR enrichment above solar R:FR, DEGs were predominantly upregulated, whereas under FR enrichment below solar R:FR, DEGs were more evenly distributed between up- and downregulation (Fig. 5b). The expression profile of genes differentially expressed in Kunta et al. (2025) and/or in this study revealed clusters with consistent up- or downregulation, while very few genes exhibited opposite responses between the two studies (Fig. 5c, Supplementary Dataset S6). The shared downregulated response was characterized by GO terms related to secondary metabolism and defense mechanisms (Fig. 5d). Gene Ontology terms related to hormonal and transcriptional regulation were present among the shared upregulated response, with three of them specifically related to auxin biosynthesis, signaling pathway, and polar transport (Fig. 5e). Among the shared light-related DEGs, *PHYTOCHROME-INTERACTING FACTOR SlPIF3* was upregulated, and *SlPIF7b* downregulated, consistent with their expression profile under prolonged darkness (Rosado et al. 2016). Moreover, *ARABIDOPSIS THALIANA HOMEOBOX2* gene homologs SlATHB2 and *ATHB2*, targets of PIF activity in tomato shade-avoidance response, were also significantly promoted by FR enrichment above solar R:FR (Supplementary Dataset S6, Kunta et al., 2025). Overall results show that leaf perception of FR rapidly upregulates auxin-responsive genes through a transient increase in IAA and initiates broader hormonal and transcriptional reprogramming. This likely represents a conserved response to a reduced R:FR, regardless of whether the R:FR ratio drops below or remains above that of solar light.

### Early fruit transcriptomic responses to Leaf FR perception involve hormone-biosynthetic genes

**Figure 6:**
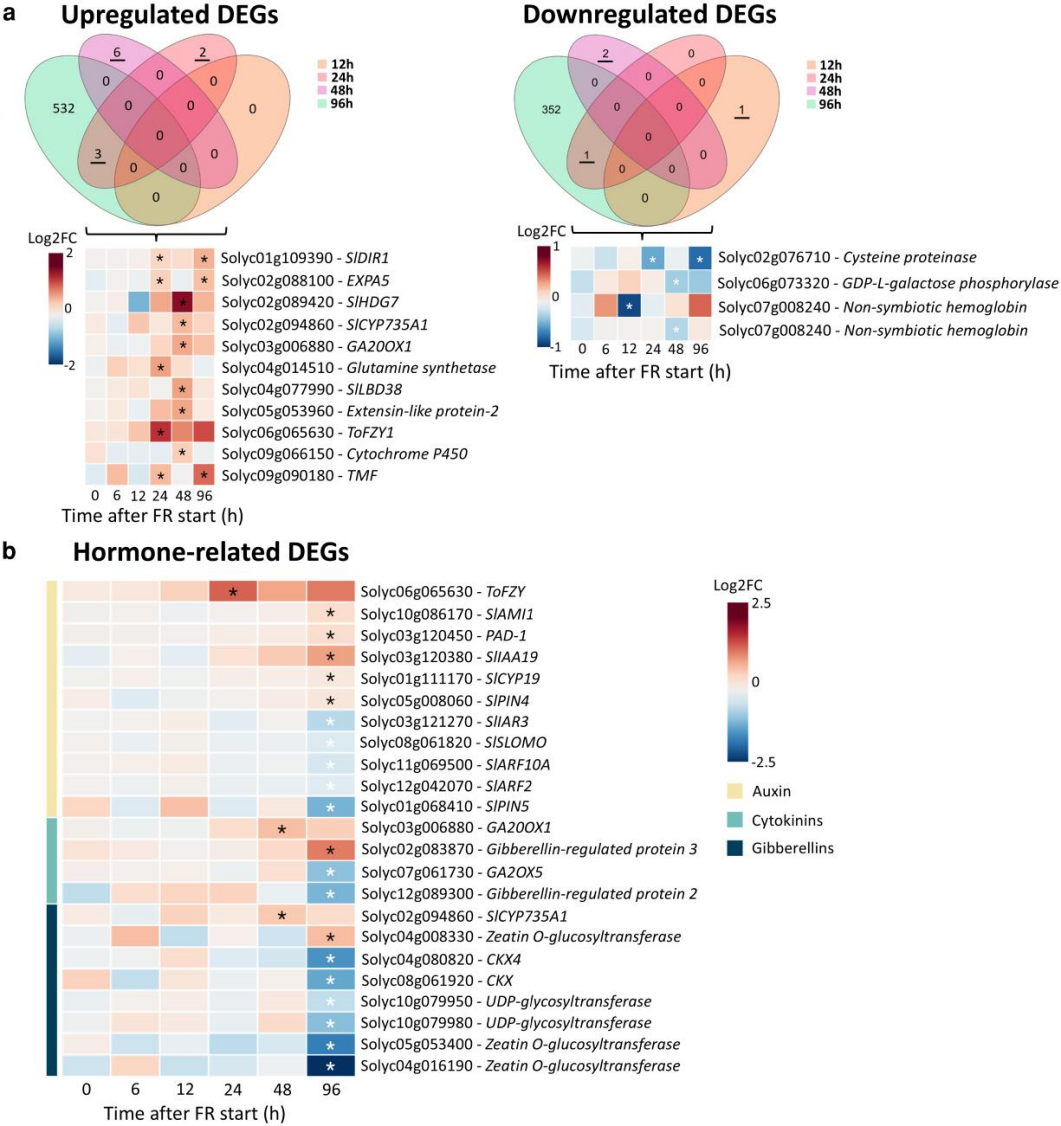
Fruit transcriptomic responses to far-red light (FR) application to vegetative organs (Leaf FR), at early time points and for hormone-related genes. Differentially expressed genes (DEGs) were identified by comparing Leaf FR vs. No FR treatments at different time points, using an adjusted *P*-value threshold of 0.05. (a) Venn diagrams show the number of up- and downregulated DEGs at each time point. Heatmaps represent the expression pattern of early responding DEGs. (b) DEGs associated with auxin, cytokinin, and gibberellin metabolism, transport, or signaling are shown in a heatmap. Significant time points (adjusted *P*-value threshold = 0.05) are indicated by asterisks on all heatmaps, with black asterisks for upregulated genes and white asterisks for downregulated genes. Complete list of abbreviations in Table S1.

As leaf responses to FR perception included a transient increase in free IAA concentration followed by prolonged upregulation of auxin-responsive genes, we further investigated all genes connected with auxin in the fruit transcriptome. Auxin is known to mediate systemic responses to FR in shade-avoidance contexts (Procko et al. 2014; Küpers et al. 2023; Gautrat et al. 2025), and it is one of the main hormones controlling early development of young tomato fruits, together with cytokinins and gibberellins (Quinet et al. 2019). Interestingly, one of the only five genes upregulated before the 48-h time point was a YUCCA-like flavin monooxygenase (*TOMATO FLOOZY 1 ToFZY1*) (Fig. 6a). Homologous to *Arabidopsis YUCCA 4 (YUC4)*, *ToFZY1* plays a key role in the tryptophan-dependent auxin biosynthetic pathway and is predominantly expressed during early fruit development in the columella tissue (Expósito-Rodríguez et al. 2007; Fernandez-Pozo et al. 2017). Auxin has been implicated in regulating the dynamics of other hormones (Mariotti et al. 2011; Liu et al. 2018), and, in our dataset, this is reflected in the subsequent upregulation of cytokinin and gibberellin biosynthetic genes at the 48-h time point, specifically *SlCYP735A1* and *GIBBERELLIN-20-OXIDASE 1* (*GA20OX1)* (Fig. 6a). Moreover, an auxin-inducible expansin precursor gene (*EXPA5*), involved in cell wall loosening and cell enlargement (Hua et al. 2024), was also present among the early upregulated genes. Despite this transcriptional evidence, we did not observe any significant change in free IAA concentration in the fruits at any of the sampled time points (Figure S5). Nevertheless, several genes associated with auxin metabolism, homeostasis, signaling, and transport were differentially expressed at the last time point (Fig. 6b), possibly indicating that auxin signaling, rather than auxin biosynthesis, would be the primary regulatory process associated with long-distance FR signaling. Additionally, DEGs involved in cytokinin and gibberellin metabolism were present at the last time point as well (Fig. 6b), showing a long-term metabolic regulation involving multiple hormones. Taken together, our data suggest that hormonal regulation represents the initial fruit transcriptomic response to Leaf FR perception, possibly mediating the following gene expression regulation.

### Fruit carbohydrate metabolism and starch accumulation increase in response to leaf perception of FR

**Figure 7:**
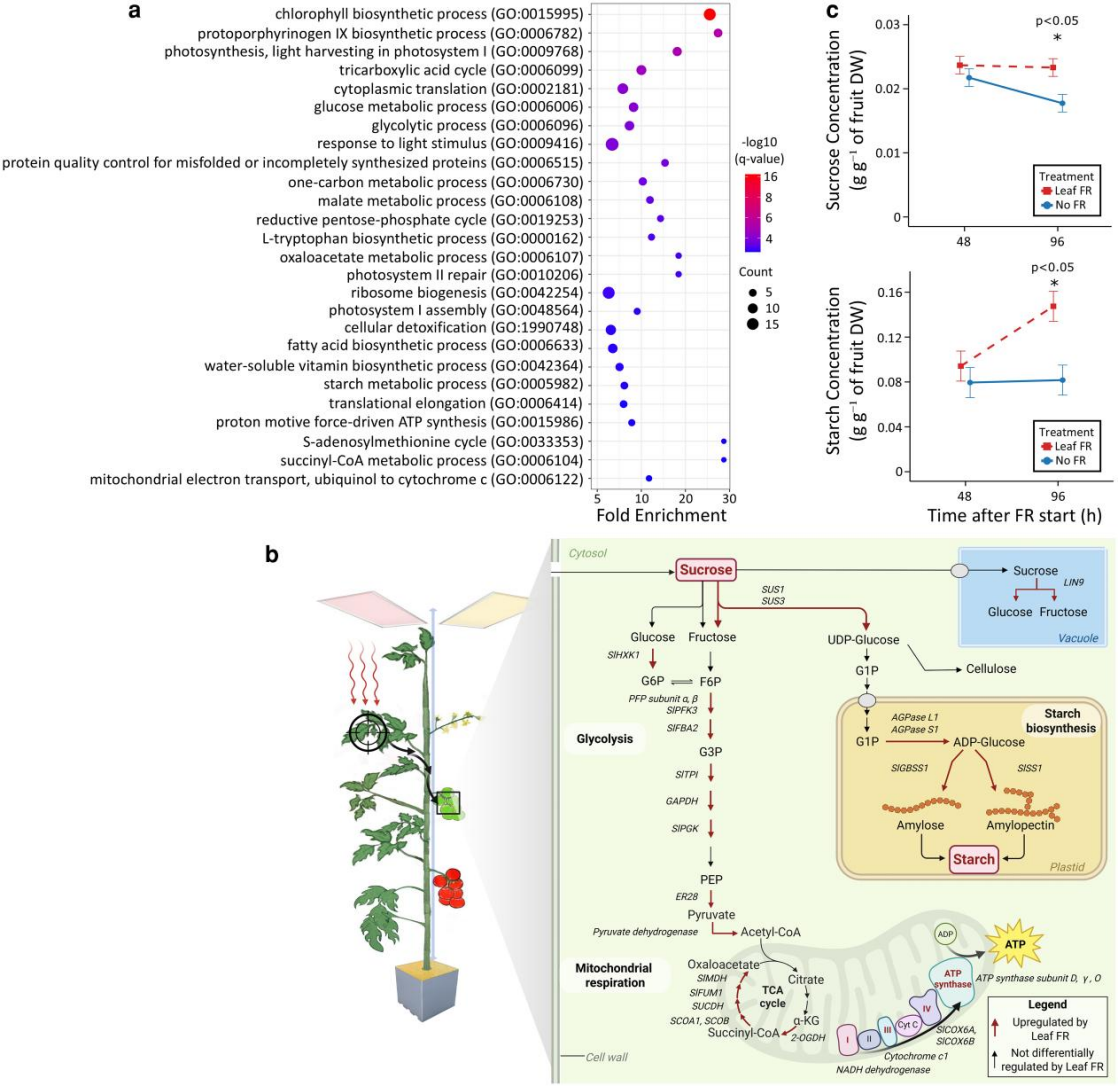
Gene Ontology (GO) enrichment, metabolic pathways, and carbohydrate accumulation in fruits following far-red (FR) light application to vegetative organs (Leaf FR). (a) Gene Ontology (GO) term enrichment analysis of upregulated differentially expressed genes (DEGs) in fruit samples 96 h after the start of Leaf FR treatment. DEGs were identified by comparing Leaf FR vs. No FR treatments, using an adjusted *P*-value (q-value) threshold of 0.05. (b) Simplified representation of starch biosynthesis, glycolysis, and mitochondrial respiration pathways upregulated in fruits at 96 h after Leaf FR treatment. Red arrows indicate reactions whose associated gene or complex subunits are upregulated, and black arrows indicate those that are not differentially regulated. Carbohydrates increased by Leaf FR in fruits at 96 h are also indicated in red. Image realized with BioRender. (c) Sucrose and starch concentrations in fruit samples at 48 and 96 h after the start of Leaf FR treatment. Data were analyzed with a two-way analysis of variance (ANOVA), with asterisks indicating significant differences according to Fisher's protected LSD test (*P* = 0.05). Values represent averages ± SEM (n = 4). α-KG, alpha-ketoglutarate; F6P, fructose-6-phosphate; G1P, glucose-1-phosphate; G6P, glucose-6-phosphate; G3P, glyceraldehyde-3-phosphate; PEP, phosphoenolpyruvate. List of abbreviations of upregulated genes in Table S2.

Since we observed increased fruit sugar concentration and dry matter partitioning to the fruits (Fig. 1d, e), we investigated whether fruit transcriptomic responses following Leaf FR perception included genes involved in sink activity and carbohydrate metabolism. Gene Ontology term enrichment analysis of upregulated DEGs in the fruit samples at the last time point (96 h after FR start, 14 days after anthesis) revealed enrichment of several categories connected with energy production and carbohydrate metabolism (Fig. 7a, Supplementary Dataset S5). In particular, multiple genes belonging to the photosynthetic machinery and the chlorophyll biosynthetic pathway were upregulated (Fig. 7a, Figure S6), suggesting a coordinated activation of chloroplast development and photosynthetic function in the fruits. Moreover, two *SUCROSE SYNTHASE* genes, *SUS1* and *SUS3*, key determinants of fruit sink activity through sucrose hydrolysis in the cytosol (Stein and Granot 2019), were upregulated in response to Leaf FR perception (Supplementary Dataset S4, Fig. 7b). Similarly, the vacuolar invertase *LYCOPERSICON ESCULENTUM INVERTASE 9 (LIN9)*, which hydrolyzes sucrose within the vacuole and thereby affects vacuolar osmolarity (Osorio et al. 2014), was also upregulated. Consistent with the gene expression data, fruit sucrose concentration at the 96-h time point was significantly higher in plants receiving the Leaf FR treatment, compared to No FR (Fig. 7c).

In young tomato fruits of most cultivated varieties, starch is transiently accumulated in plastids of the inner pericarp and then remobilized later during fruit development (Schaffer and Petreikov 1997). Transient starch accumulation arises largely from elevated starch biosynthetic activity, rather than reduced degradation (Robinson et al. 1988), and the first committed step is catalyzed by ADP–glucose pyrophosphorylase (AGPase), converting glucose-1-phosphate (G1P) into ADP–glucose (ADPG) (Schaffer et al. 2000). ADP–glucose pyrophosphorylase is a tetrameric enzyme whose long and short subunits (*AGPase L1* and *S1*) were upregulated in response to Leaf FR, along with *SOLUBLE STARCH SYNTHASE* and *GRANULE-BOUND STARCH SYNTHASE* genes (*SlSS1* and *SlGBSS1*, respectively) (Supplementary Dataset S4, Fig. 7b). Starch synthases use ADPG to elongate amylopectin and amylose chains, the two main glucose polymers constituting starch. As starch concentration in young fruits positively correlates with soluble sugar concentration in ripe fruits (Robinson et al. 1988; Schaffer and Petreikov 1997), we quantified the starch levels in our fruit samples. Fruit starch concentration of Leaf FR-treated plants significantly increased between 48 and 96 h, exceeding No FR levels at 96 h and indicating an earlier onset of starch accumulation (Fig. 7c). Together with the upregulation of several genes involved in glycolysis, tricarboxylic-acid cycle, and mitochondrial electron transport chain (Fig. 7a), these results strongly suggest that Leaf FR perception leads to increased sucrose import and cleavage into hexoses, fueling starch accumulation and energy production to support fruit growth.

The downregulated gene set in fruit tissue was enriched (adjusted *P*-value threshold = 0.05) for GO categories related to self-incompatibility, carbohydrate transport, and response to wounding (Figure S7, Supplementary Dataset S5). We further investigated the genes related to “carbohydrate transmembrane transport.” Downregulated carbohydrate transporters belonged mostly to the Sugars Will Eventually be Exported Transporter (SWEET) and Sugar Transporter Protein (STP) family, both playing a role in the apoplastic sucrose unloading pathway (Reuscher et al. 2014; Milne et al. 2018). During the early stages of fruit development, sucrose is predominantly unloaded into the pericarp via a symplastic route, enabled by direct cell-to-cell connections between phloem elements and vascular and storage parenchyma cells (Ma et al. 2019). Apoplastic unloading, requiring sucrose and hexose transporters, assumes a predominant role at a later stage of fruit development, but it is still present to a lesser extent in early developing fruits (Beckles et al. 2012; Ko et al. 2021). Therefore, we concluded that the downregulation of sucrose and hexose transporters at 96 h is likely a consequence of increased sucrose import triggered by Leaf FR treatment, which occurs predominantly via the symplastic route.

## Discussion

The addition of FR to PAR light increases individual fruit weight and enhances soluble sugar concentration (Fanwoua et al. 2019; Ji et al. 2020; Vincenzi et al. 2024). However, it remains unclear whether local perception of FR light within the fruits is required to trigger fruit growth responses. In this study, we controlled the light environment of the vegetative and generative organs of tomato plants and showed that FR light perception by vegetative organs mediates fruit growth and metabolic responses to FR.

### FR-mediated increase in fruit sink strength requires interorgan signaling

Fruit growth largely depends upon the size of the plant assimilate pool and the fruit's competitive ability to attract assimilates, as described by the source–sink model for plant resource allocation (Fernie et al. 2020). In this context, a source tissue is a net producer of assimilates, while a sink tissue is a net importer. A higher source strength increases the assimilate pool, thereby enabling an increase in the growth of all sinks in absolute terms. Conversely, greater fruit sink strength shifts assimilate allocation in favor of the fruits (Marcelis 1996). Our study confirms that FR perception by the vegetative organs is both necessary and sufficient to achieve the full effect of FR on plant dry weight, aligning with the FR-induced increase in leaf CO_2_ assimilation rate (Figure S3). The increase in plant dry weight was caused by an increase in fruit dry weight, as the dry weight of the vegetative organs was similar across all treatments (Fig. 1c). Far-red enrichment enhanced the fraction of dry matter partitioned to the fruits (+18% Full FR vs. No FR) and stem, at the expense of the leaves (Fig. 1d). Importantly, the increase in the fraction of dry matter partitioned to the fruits was driven by FR perception in the vegetative organs, rather than by FR supplied directly to the fruits (Fig. 1d). Dry matter partitioning is not influenced by the plant source strength, as shown in tomato by altering the intensity of intercepted PAR (Heuvelink 1995; Schouten et al. 2024). In particular, under FR enrichment, higher dry matter partitioning to the fruits is determined by an increase in individual fruit sink strength, rather than a decrease in the strength of competing sinks (Ji et al. 2020; Vincenzi et al. 2024). Individual fruit sink strength can be quantified by potential fruit growth under unlimited assimilate supply, and it is therefore also independent of the plant source strength (Marcelis 1996). For instance, more than doubling incident PAR, from 300 to 700 μmol m^−2^ s^−1^, increased plant dry weight without altering the individual fruit sink strength in dwarf tomato (Ke et al. 2023). In conclusion, while the increase in plant dry weight under FR enrichment can be explained by enhanced photosynthesis, the shift in dry matter allocation toward the fruits is likely a photomorphogenic response to a decrease in the R:FR ratio perceived by the vegetative organs. As fruit sink strength is determined locally within the fruit tissue, we propose that the FR-induced increase in dry matter partitioning to the fruits, due to stronger fruit sink strength, represents a systemic response. This response involves communication between the vegetative organs perceiving the FR signal and the fruiting trusses where the response is ultimately expressed.

### Far-red perception by the vegetative organs promotes fruit growth through enhanced sink strength

Our transcriptomic analysis showed that FR perception by the vegetative organs led to an upregulation of genes associated with both source and sink activity in the fruits (Fig. 7a, b). Green tomato fruits are photosynthetically active, and estimates suggest that fruit photosynthesis accounts for 10% to 15% of fruit dry weight (Tanaka et al. 1974). Nevertheless, the contribution of fruit photosynthesis to fruit growth remains under debate, as the lack of in situ photosynthesis can be compensated by increased import of sucrose through the phloem, without affecting fruit weight (Lytovchenko et al. 2011; Okello et al. 2015). Following FR perception by the vegetative organs, expression of genes related to sucrose import and cleavage into early developing fruits increased (Fig. 7b). This was highlighted by the upregulation of *SUS1* and *SUS3* and by the higher sucrose concentration in Leaf FR fruits 96 h after the start of the FR treatment, compared to the No FR fruits (Fig. 7b, c). Six sucrose synthases have been described in tomato, and among them, *SUS1* is considered the prevalent one, influencing sucrose unloading into the fruits and consequently promoting fruit set and fruit growth rate (D’Aoust et al. 1999). Despite the upregulation of genes associated with sucrose import, the corresponding levels of glucose and fructose in the fruits were not increased upon FR enrichment. Together with the upregulation of many genes belonging to the glycolysis and mitochondrial respiration pathways, this suggests that FR promotes a higher rate of hexose metabolism to support growth in an energy-intensive period of fruit development, when fruit growth rate is close to its maximum (Ji et al. 2020). At this stage, a rapid increase in fruit weight is strongly determined by cell expansion (Cheniclet et al. 2005), which in turn is driven by water inflow triggered by increasing cell osmotic potential. This increase in cell osmotic potential is induced by the accumulation of soluble sugars and organic acids sequestered in the vacuole. Vacuolar acid invertases, such as *LIN9*, which was upregulated in Leaf FR fruits at 96 h, contribute to this process by cleaving sucrose into glucose and fructose inside the vacuole (Osorio et al. 2014). Moreover, FR perception by the vegetative organs resulted in upregulation of key components of the starch biosynthetic pathway, in particular the rate-limiting *AGPase*, resulting in increased starch concentration in fruits 96 h after the start of the FR treatment (Fig. 7b, c). Increasing AGPase activity, and therefore starch accumulation, in developing tomato fruits was correlated with higher soluble solids content and dry weight in ripe fruits, making this an important mechanism to determine fruit sink strength (Petreikov et al. 2006).

While our results were obtained by localized FR enrichment of the vegetative organs, similar findings were reported following whole-plant FR exposure (Ji et al. 2020). The authors reported that fruit growth rate, starch accumulation, and sink activity in young tomato fruits were increased by whole-plant FR enrichment, leading to higher fruit weight at the ripe stage. The fact that comparable effects were achieved when only the vegetative organs were exposed to FR further supports our hypothesis that vegetative organs are the primary site responsible for mediating fruit growth responses to FR. Tomato fruits express phytochromes, and fruit-localized knockout or constitutive expression of these photoreceptors, in particular of the most abundant *SlPHYB2* (Bianchetti et al. 2017), affected several processes, among which are plastid biogenesis and carotenoid metabolism (Ernesto Bianchetti et al. 2018; Alves et al. 2020). However, in our study, FR enrichment of generative organs did not alter fruit weight, fruit growth period, sugar concentration, or composition on its own (Figs. 1, 2b, Figure S2). Nevertheless, the plants that received FR enrichment of both vegetative and generative organs had a significantly higher sugar concentration in ripe fruits than any other treatment (Fig. 1e), suggesting that fruit-localized perception of FR can promote sugar accumulation, but only when the vegetative organs are also exposed to FR.

### Hormone signaling as a candidate mechanism linking far-red perception to fruit responses

Following the start of the Leaf FR treatment, the genes showing the earliest differential expression suggest altered fruit hormonal metabolism through enhanced biosynthesis of auxin, GAs, and cytokinins (Fig. 6a). Interestingly, auxin and cytokinin signaling have been linked to the regulation of chlorophyll accumulation and carbohydrate dynamics in the fruits of the phytochromobilin-deficient *aurea* mutant. In this mutant, reduced auxin and cytokinin signaling were associated with lower chlorophyll and chloroplast abundance, decreased starch accumulation, and reduced soluble sugar levels (Bianchetti et al. 2017). In our study, FR exposure triggered a rapid but transient increase in free auxin concentration in the leaves, followed by long-term regulation of auxin-responsive genes (Fig. 4). Auxin plays a key role in both early fruit development (Quinet et al. 2019) and interorgan signaling following FR perception (Procko et al. 2014; Iglesias et al. 2018; Küpers et al. 2023; Oskam et al. 2024). However, no differences in auxin concentration were detected between fruit samples from FR-treated and control plants. Nevertheless, auxin involvement in fruit responses to FR cannot be ruled out. Previous work has suggested that auxin response, rather than auxin content, is the primary link between phytochrome and auxin pathways in developing fruits (Bianchetti et al. 2017), and we observed several genes associated with auxin response and sensitivity being upregulated in response to Leaf FR (Fig. 6b). Cytokinins have also been implicated in interorgan FR signaling (Gautrat et al. 2024). In tomato, cytokinins influence fruit size by regulating cell division, but they also control chloroplast biogenesis and division (Gan et al. 2022). For example, increased chloroplast number and chlorophyll accumulation in the *high pigment 1* (*hp1*) mutant were linked to altered cytokinin sensitivity (Caspi et al. 2008). Finally, exogenous cytokinin application has been shown to promote sink strength by increasing sucrose synthase and invertase activity in tomato fruits (Albacete et al. 2014), making this hormone an interesting candidate for driving fruit growth responses to FR. Future research aiming to resolve the mechanism mediating interorgan FR signaling to the fruit and initiating fruit growth responses should therefore examine the potential role of plant hormones, particularly auxin and cytokinins.

### Transcriptomic and hormonal dynamics reveal a conserved response to decreasing R:FR

In this study, leaf auxin content and transcriptomic regulation peaked at the earliest measured time point (6 h) following the start of the FR treatment (Figs 3b, 4c). However, fruit dynamics in response to vegetative organ perception of FR were different, showing no increase in fruit auxin content and a delay of 96 h in the onset of the fruit transcriptomic response and changes in carbohydrate levels (Figure S5, Figs 3c, 7c). Similar dynamics between vegetative and generative organs were reported in *Arabidopsis* under light conditions where shade was simulated by strong FR enrichment in a white light background (Roig-Villanova et al. 2025). In that study, FR enrichment below solar R:FR increased auxin levels in leaves but not in inflorescences and reduced the ovule number with a delay of 2 to 4 days from the start of the FR treatment. These similarities suggest a conserved response to decreasing R:FR ratios, regardless of whether they remain above solar R:FR or not, aligning with the high similarity in the leaf transcriptomic response across these two conditions (Fig. 5). Despite using a different R:FR window, always above solar levels, we identified a similar response as observed for R:FR ratios that induce shade avoidance (Kunta et al. 2025), including the upregulation of genes involved in transcriptional and hormonal regulation, in particular regarding auxin, brassinosteroids, and cytokinins. Also, we observed the shared downregulation of defense mechanisms and secondary metabolite biosynthesis, particularly anthocyanins and steroidal glycoalkaloids (Supplementary Dataset S6). Finally, our response to FR enrichment in leaves appears to share at least part of the photomorphogenic pathway typical of shade avoidance, as we found multiple PIFs, PIF targets, and the light-signaling messenger ELONGATED HYPOCOTYL 5 (HY5) among the differentially regulated genes (Supplementary Dataset S6). It remains to be determined whether the similarity observed in leaf response to FR enrichment, above or below solar R:FR, could apply to the generative organs as well. For instance, while FR enrichment below solar R:FR reduces seed number per silique (Roig-Villanova et al. 2025), seed number per fruit is not affected by FR enrichment above solar R:FR (Ji et al. 2020).

## Conclusions

We showed that tomato fruit growth responses to FR enrichment depend on FR perception in the vegetative organs, including leaves, stem, and apex, rather than flowering and fruiting trusses. Locally, FR exposure of the vegetative organs triggered a transient rise in free auxin concentration, with a sustained activation of auxin-responsive genes, highlighting a conserved response to decreasing R:FR ratios. Systemically, FR enrichment promoted sink-related gene expression, sucrose import, and starch accumulation in immature green fruits, leading to higher fruit weight and soluble sugar levels in ripe fruits. Far-red-induced increase in assimilate partitioning toward the fruits is a systemic response that requires interorgan communication.

### Materials and methods

This study involves two experiments: In Expt. 1, conducted in 2023, data were collected on red ripe fruit weight, dry matter content, and sugar concentration. In Expt. 2, conducted in 2024, data were collected on gene expression, starch and sugar concentration, and auxin concentration in leaves and early developing fruits following the start of the FR treatment.

### Plant material and growth conditions

In both experiments, seeds of the commercial cluster tomato hybrid *Solanum lycopersicum* L. cv. Foundation (BASF—Nunhems, Nunhem, The Netherlands) were sown in stonewool starter plugs (Grodan, Roermond, the Netherlands) in a greenhouse compartment at Wageningen University and Research (52°N, 6°E, Wageningen, the Netherlands). Seeds were sown on 4 April 2023 (Expt. 1) and on 20 June 2024 (Expt. 2). Two weeks after emergence, the seedlings were placed into 15 × 15 cm stonewool blocks (HUGO blocks, Grodan). Uniform plants were selected and transplanted to a climate chamber (500 × 300 × 250 cm; length × width × height) 4 (Expt. 1) or 5 (Expt. 2) weeks after sowing. The climate chamber was divided into 14 experimental units, each measuring 150 × 30 × 230 cm, separated by light-impermeable white plastic sheets. Each unit included a gutter with four plants. Airflow was directed from one long side of the chamber to the other. In Expt. 2, an additional fan (12 × 12 cm, 4414 N, ebm-papst, Mulfingen, Germany) was added to each unit to increase ventilation below the canopy, and the fan height was adjusted as the plants grew.

In both experiments, plants were irrigated using a drip system with a standard nutrient solution for tomato fertigation with an electric conductivity of 2.0 dS m^−1^ and a pH of 6.0 (Table S3). Average air temperatures were 21.7 ± 0.2 °C during the photoperiod (16 h) and 19.1 ± 0.1 °C during the dark period in Expt. 1 and 21.0 ± 0.1 °C and 18.1 ± 0.1 °C in Expt. 2. Average daily relative humidity (24 h) was 71 ± 4% in Expt. 1 and 77 ± 1% in Expt. 2, and the daily CO_2_ concentration was 404 ± 30 µmol mol^−1^ in Expt. 1 and 426 ± 43 µmol mol^−1^ in Expt. 2. Each plant was supported by a wire connected to the top of the climate chamber, according to high-wire growing practices. Lamp height was adjusted weekly to maintain a minimum distance of 30 cm between the top of the plants and the lamps. In Expt. 1, 94 days after sowing (DAS), plants reached the maximum height allowed by the chamber setup, and plant growth was arrested by removing the apex. The first flowering truss was pruned to two flowers, and all subsequent trusses were pruned to four flowers each, to maintain a reasonable balance between assimilate production in leaves (source) and assimilate demand for fruit development (sink). Pollination was carried out manually three times per week with a Vibri Vario electronic bee (Royal Brinkman, “s Gravenzande, the Netherlands).

### Light conditions and far-red treatments

In Expt.1, photosynthetic photon flux density (PPFD) at the top of the canopy started at 150 ± 3 µmol m^−2^ s^−1^ upon transplant into the climate chamber, and it gradually increased together with plant height until it reached the intensity of 215 ± 4 µmol m^−2^ s^−1^, which was maintained from 79 DAS until the end of the experiment. Photosynthetic photon flux density was provided by LEDs with adjustable spectrum mounted on the ceiling of the chamber (Green Power Dynamic 2.0 LED research modules with a GrowWise Control System, Philips, Eindhoven, the Netherlands), resulting in a RW spectrum of R:G:B:FR 80:3:16:1 (Figure S8). Far red for treatments was provided by one additional LED module per experimental unit (79 ± 1 µmol m^−2^ s^−1^, GreenPower LED2.2 FR 150 RO, Philips). Four light treatments were applied: FR was not supplemented to any part of the plant (No FR); supplemented only to the vegetative organs (Leaf FR); only to the generative organs (Fruit FR); and to both the vegetative and generative organs (Full FR) (Table 1). The vegetative organs of each plant included leaves, stem, apex, and pre-anthesis trusses, while the generative organs included flowering trusses with at least one flower fully open and fruiting trusses. In the experimental units where FR was not supplemented, dummies were used to simulate the shading from the additional FR modules. Red and white and FR lighting followed the same photoperiod of 16 h of light per day, from 4 AM to 8 PM.

In Expt. 2, only two light treatments were applied: No FR and Leaf FR (Table 1). All treatments received a PPFD of 258 ± 5 µmol m^−2^ s^−1^, which was supplied from transplant to the end of the experiment by RW modules (R:G:B 76:1:23, GreenPower Toplight DR/W MB, Philips). Far red (77 ± 1 µmol m^−2^ s^−1^) was supplied as in Expt. 1.

In Expt. 1, the FR treatment started at anthesis of the first flower on the second truss, 59 DAS. In Expt. 2, the FR treatment started 10 days after anthesis of the first flower of the second truss, 63 DAS. At the start of the FR treatment, plants were rotated such that their first and second trusses faced one of the plastic sheets delimiting their experimental unit. Openings were cut in the sheet, trusses were passed through, and cuts were sealed with tape. To train plants against the sheet, every third leaf growing toward it was removed. Afterward, each new truss reaching anthesis was put through the curtain when possible, and one leaf below the truss developing in the direction of the curtain was removed. On average, one truss per plant was not passed through the curtain; these trusses were excluded from any analysis, except the final destructive plant harvest.

### Plant measurements

#### Growth and development measurements

The number of leaves and stem length were assessed weekly until 69 DAS (end of Expt. 2) and 96 DAS (plant decapitation in Expt. 1). Dry weight of all pruned leaves was recorded per experimental unit and used to calculate the total plant weight. The number of open flowers in each flowering truss was recorded three times per week throughout the experiment and used to calculate the flowering rate.

#### Fruit ripening and harvest

In Expt. 1, fruit ripening rate was recorded three times per week until the end of the experiment. Ripening was monitored from “breaker stage” until “light-red stage” (USDA, 1991) using a portable colorimeter Minolta CR-400 (Konica Minolta, Osaka, Japan) to measure fruit pericarp color. Fruits at the “breaker stage” were identified by visual inspection, and fruit ripening was assessed with three measurements on the fruit equatorial side. A fruit was considered ripe and harvested when the average a* value of the three measurements reached a threshold value of 16, with no individual measurements below 14. The threshold value was determined for this cultivar by correlating visual scores with colorimeter measurements (Figure S9). Fruit fresh weight was recorded before cutting each tomato into two halves. One-half was immediately frozen in liquid nitrogen for carbohydrate quantification, while the fresh and dry weights of the other half were recorded before and after oven-drying for 48 h at 105 °C for dry matter content determination. Dry matter content was then used to calculate the dry weight for the whole fruit. A total of 296 fruits from the first and second trusses of each plant were individually harvested and measured.

#### Plant destructive harvest

Destructive plant measurements were carried out at transplant (baseline measurements) and at 132 DAS in Expt. 1 and 69 DAS in Expt. 2 (final plant harvest). In Expt. 1, internode length between nodes 15 (start of FR treatment) and 25 (plant decapitation) was measured, along with the number of leaves (length ≥ 2 cm), number of trusses (at least one fully open flower), and fruit number (fruit diameter ≥ 2 cm). Average leaf area was measured for five randomly selected leaves per plant using a leaf area meter (LI-3100 area meter, Li-Cor). Aerial plant organs were separated into stem, leaves, and fruits and dried separately at 70 °C for 50 h and then at 105 °C for 75 h before determining their dry weight. Total plant dry weight is the dry weight determined at the destructive harvest, added to the dry weight of harvested ripe tomato fruits and pruned leaves. In Expt. 2, six plants per treatment were randomly selected, and the number of leaves, total leaf area, and plant dry weight were measured.

### Carbohydrate extraction and quantification

Fruit samples were frozen in liquid nitrogen, freeze-dried for 5 days (Alpha 1 to 4 LSCbasic, Salm en Kipp, Breukelen, the Netherlands), and then ground into fine powder using a mortar and pestle. In Expt. 1, equal weights of fruit samples from the same truss and plant within each experimental unit were pooled together, mixed well, and used for carbohydrate quantification. In Expt. 2, carbohydrate quantification was carried out for fruit samples collected at 48 and 96 h after the start of the FR treatment, individually. Starch, glucose, fructose, and sucrose concentrations were quantified as described by Ji et al. (2020), with the same equipment and minor adjustments: 15 mg of the freeze-dried samples were weighed and used for the extraction, and samples were diluted with MilliQ water before quantification (Full method: Supplementary Method S1).

### Gene expression analysis

#### Sample collection

Leaf and fruit samples were collected at six time points: 0 (pre-FR exposure), 6, 12, 24, 48, and 96 h after the start of the FR treatment. Fruit samples consisted of the first fruit from the second truss at the “immature green” stage (10 ± 1 days after anthesis). Leaf samples consisted of the right leaflet from the most distal pair on the leaf above the second truss (leaf 13, when leaf 0 is the first true leaf). Four plants per treatment were sampled per time point, with no plant harvested twice or from the same unit at the same time point. Samples were immediately labeled and frozen in liquid nitrogen, with all samples for each time point harvested within 10 min. Frozen samples were ground into fine powder with a mortar and pestle.

#### RNA sequencing, alignment, and functional data analysis

A total of 96 ground, frozen leaf and fruit samples were sent to BGI Genomics (Hong Kong) for RNA extraction and RNA-seq analysis, accounting for two light treatments, two organs, six time points, and four biological replicates (Supplementary Dataset S1). All RNA samples had RNA integrity (RIN) values ≥ 6.0, a total RNA ≥ 400 ng, and 28S/18S ≥ 1.0, except for one sample that was excluded from further analysis. Library preparation and sequencing were performed by BGI using the DNA Nanoball Sequencing (DNBSEQ) platform with PE150. Quality control and filtering of raw sequencing data and alignment were carried out by BGI (Full pipeline: Supplementary Method S2). Tomato genome ITAG4.0 (Hosmani et al. 2019) was used as reference. Raw read counts are provided in Supplementary Dataset S2, and normalized read counts used for downstream analyses are provided in Supplementary Dataset S3. Differentially expressed genes were identified using an adjusted *P*-value (Benjamini–Hochberg q-value) threshold of 0.05, and GO enrichment analysis of the DEG sets was carried out using Fisher's exact test with a q-value threshold of 0.05 with PANTHER (PANTHER version 19.0). The clustered heatmap of genes highly differentially expressed among the leaf transcriptomic response to 6 h of FR enrichment in this study and/or in Kunta et al. (2025), but present in both, was done using the R package pheatmap (version 1.0.13). Data visualization has been carried out with RStudio (software version 4.4.1, Rstudio, PBC, Boston, MA), BGI data visualization system (Dr. Tom Data Visualisation Solution), and BioRender (BioRender).

### Auxin extraction and quantification

The same 96 samples used for RNA extraction and sequencing were used for auxin extraction and quantification. Free auxin (indole-3-acetic acid, IAA) was extracted and quantified as previously described (Ruyter-Spira et al. 2011; Xiao et al. 2025). In short, 20 mg of ground, frozen tissue was extracted in 1 mL of cold methanol containing [phenyl ^13^C_6_]-IAA (0.1 nmol/ml) overnight at 4 °C. During the extraction procedure, samples were kept on a shaker and in the dark. After extraction, samples were centrifuged at full speed for 10 min at 4 °C. Indole-3-acetic acid was extracted from the supernatant using a solid phase extraction cartridge Oasis® MCX 1CC/30 mg (Water Corporation, Oud-Gastel, the Netherlands), filtered through a 0.45 μm Minisart SRP4 filter (Sartorius, Goettingen, Germany), and quantified by multi-reaction monitoring liquid chromatography–tandem mass spectrometry (MRM-LC-MS/MS).

### Experimental design and statistical analysis

Expt. 1 consisted of 14 experimental units of four plants each, divided among four treatments (n = 3 for Fruit FR and Leaf FR, n = 4 for No FR and Full FR). Data were analyzed using a one-way ANOVA, with light treatment as the main factor. For parameters related to individual ripe fruit, namely, fruit weight, fruit dry matter content, fruit sugar content, and fruit growth period, variation due to biological differences between different trusses was accounted for by including the truss number as a blocking factor. For two experimental units (not in the same treatment), PPFD was 10% to 15% lower at the top of the canopy due to climate chamber setup limitations. Therefore, PPFD measured at the top of the canopy of each unit was included as a covariate in the ANOVA. Expt. 2 consisted of 12 experimental units of four plants each, equally divided among two treatments (No FR and Leaf FR). A time course was conducted by sampling leaves and fruits from four plants per treatment at six time points (n = 4). Data were analyzed according to a completely randomized design.

Statistical analyses were performed with Genstat (24th Edition, VSN International, London, UK), and data visualization and graphs were realized with RStudio. The assumption of normal distribution and homogeneity of variance were assessed and confirmed via the Shapiro–Wilk test (*P* > 0.05) and Levene test (*P* > 0.05). Fisher's protected LSD test (*P* = 0.05) was used for mean separation.

### Accession number

The RNA sequencing data discussed in this publication have been deposited in NCBI's Gene Expression Omnibus and are accessible through GEO Series accession number GSE320482

(https://www.ncbi.nlm.nih.gov/geo/query/acc.cgi?acc  =  GSE320482).

## Supplementary Material

kiag358_Supplementary_Data

## Data Availability

The RNA sequencing data underlying this article are available in NCBI's Gene Expression Omnibus, and can be accessed through GEO Series accession number GSE320482. Other data are available on request.
