# Supplementary material for: Far-red perception by vegetative organs and not fruits drives fruit growth responses in tomato plants
Source: Plant Physiol. 2026 Jun 15;201(3):kiag358. doi: 10.1093/plphys/kiag358 (PMC13360295; doi:10.1093/plphys/kiag358)
Supplement: kiag358_Supplementary_Data [file kiag358_supplementary_data.zip › Supplementary Data.pdf]

Supplementary Data

Supplementary Figures

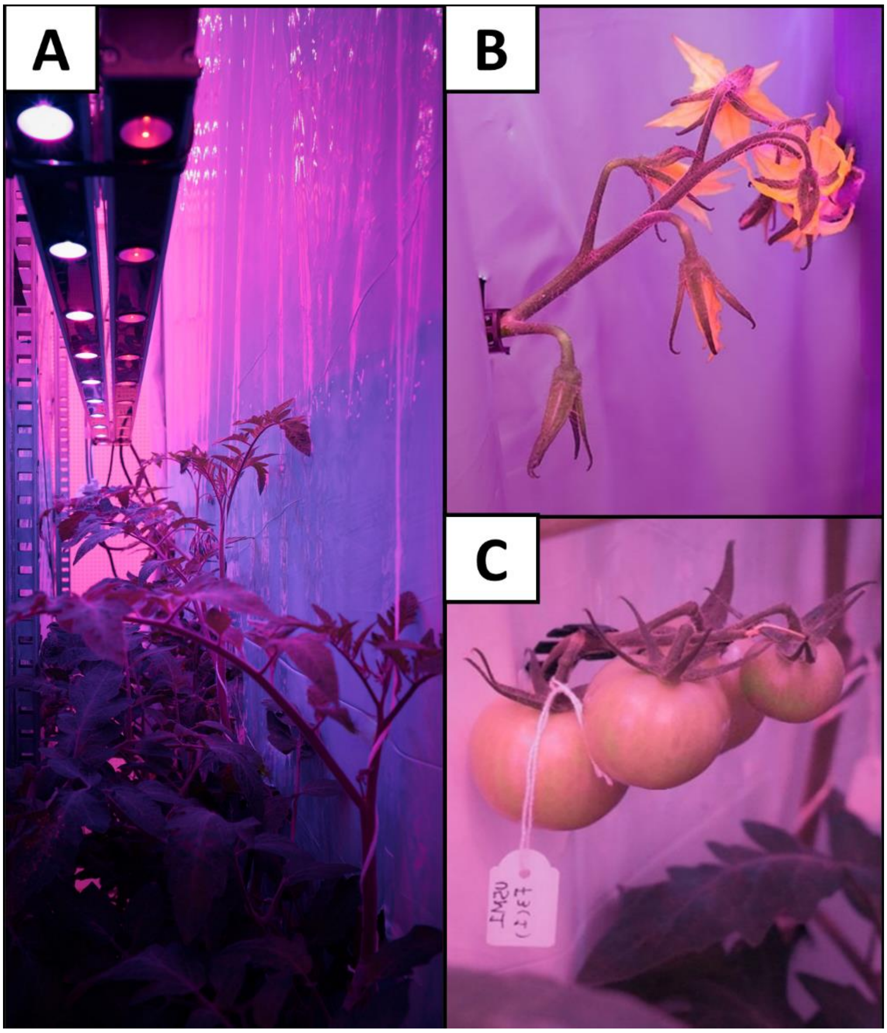

Supplementary Figure S1. Picture of experimental setup. (A) Vegetative organs of the plants (stem, leaves, and apex) are grown under separate light treatments compared to the generative organs of the plants ((B) flowering trusses and (C) fruiting trusses). A light-impermeable curtain is used to separate the light treatments.

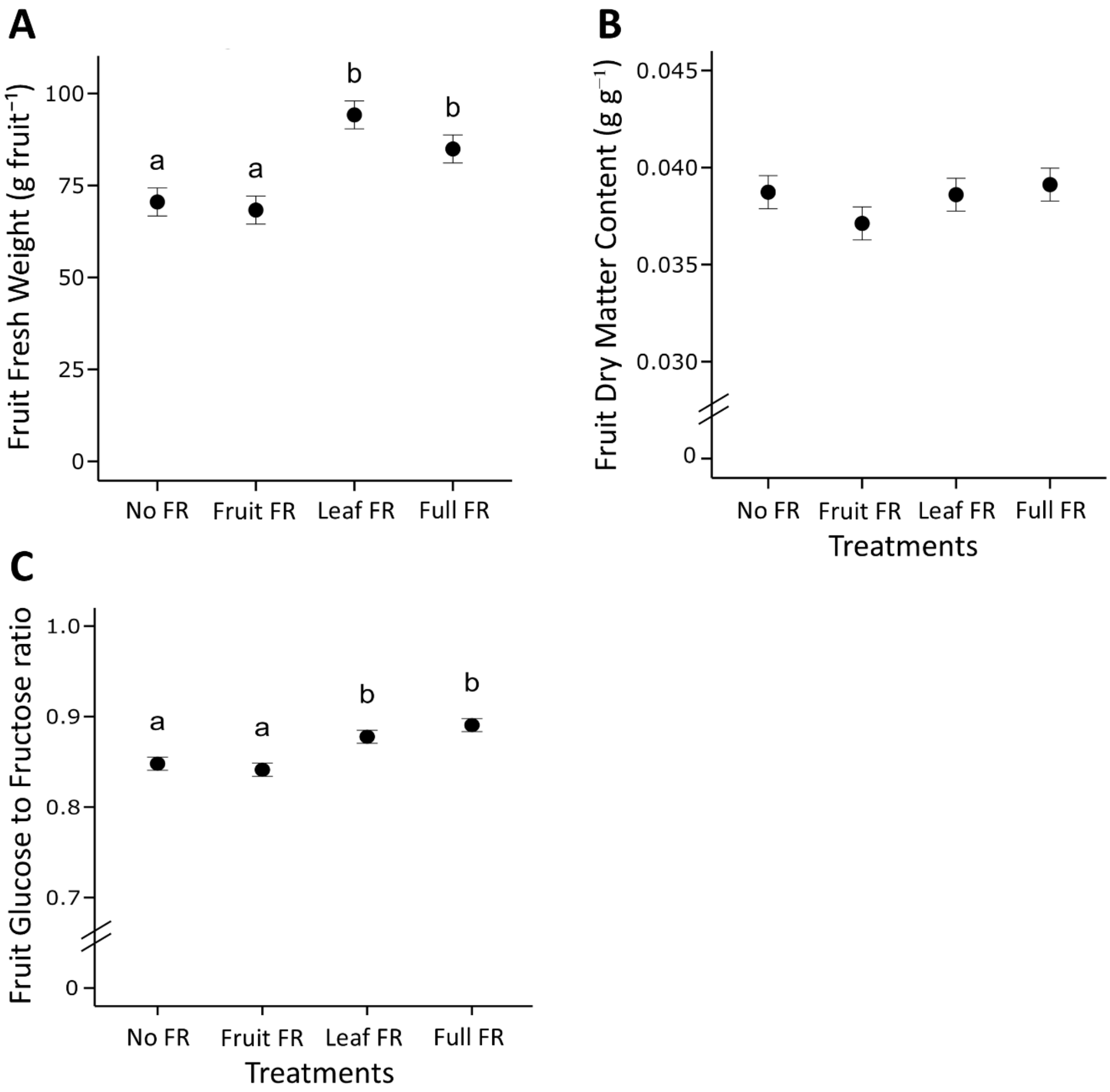

Supplementary Figure S2. Effects of far-red light (FR) treatments on ripe fruit parameters. (A) Individual fruit fresh weight. (B) Fruit dry matter content. (C) Fruit Glucose to Fructose ratio. Values are averages of two fruit trusses. Different letters indicate significant differences according to Fisher's protected LSD test (p-value threshold of 0.05). Values represent the average of 3 to 4 experimental units ± SEM (n = 3 for Fruit FR and Leaf FR, n = 4 for No FR and Full FR), each consisting of 4 plants.

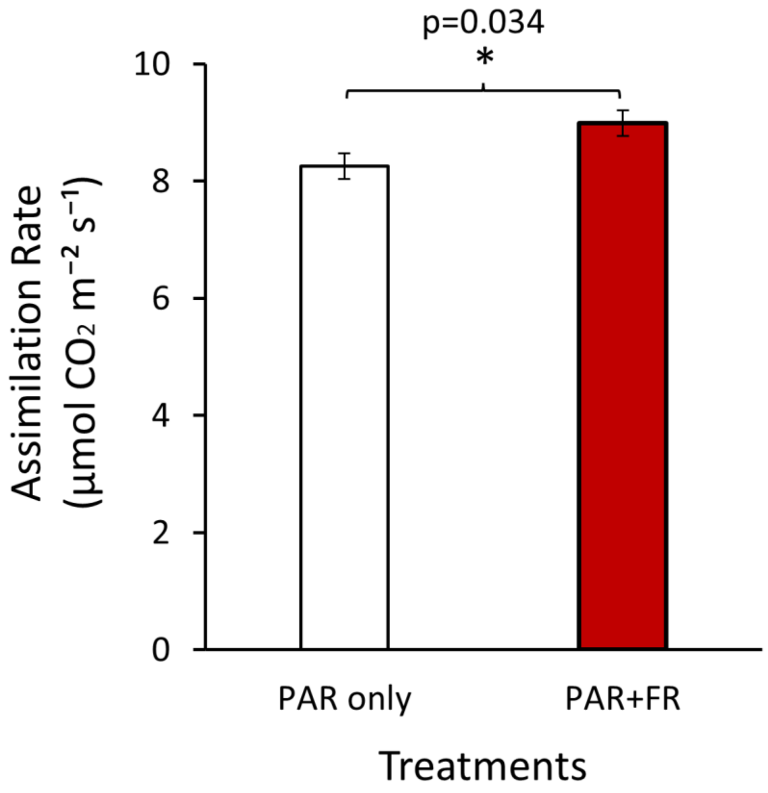

Supplementary Figure S3. Effect of the addition of far-red light (FR) on the leaf assimilation rate of tomato plants, cv. Foundation. Columns represent the average of 9 measurements, each representing a leaf from a distinct plant ± SEM. Statistical difference between the two was tested with a t-test. The white column represents the leaf assimilation rate for plants grown and measured under PAR only (200 µmol m^{-2} s^{-1}, R:B 95:5, R:FR 122). The red column represents the leaf assimilation rate for plants grown and measured with PAR + FR (FR = 30 µmol m^{-2} s^{-1}, R:FR 6, PAR+FR).

Measurements were carried out on the 4th or 5th fully expanded leaf from the apex of tomato plants grown in a greenhouse compartment during the winter months (September 2023 – February 2024) under supplementary PAR lighting (250 µmol m^{-2} s^{-1}, R:G:B 88:5.5:5.5), without (PAR only) or with additional FR (FR = 60 µmol m^{-2} s^{-1}, PAR+FR). Leaf CO_2 assimilation rate was measured on detached leaves kept in water, with the Li-6400 photosynthesis system (Li-Cor Biosciences, Lincoln, NE, USA) equipped with a clear-top chamber under PAR light (200 µmol m^2 s^{-1}, R:B 95:5, R:FR 122), without (PAR only) or with additional FR (30 µmol m^{-2} s^{-1}, R:FR 6, PAR+FR). Internal chamber parameters were standardized at a temperature of 22°C, 65% relative humidity, a flow rate of 400 µmol s^{-1}, and a CO_2 concentration of 400 µmol mol^{-1}.

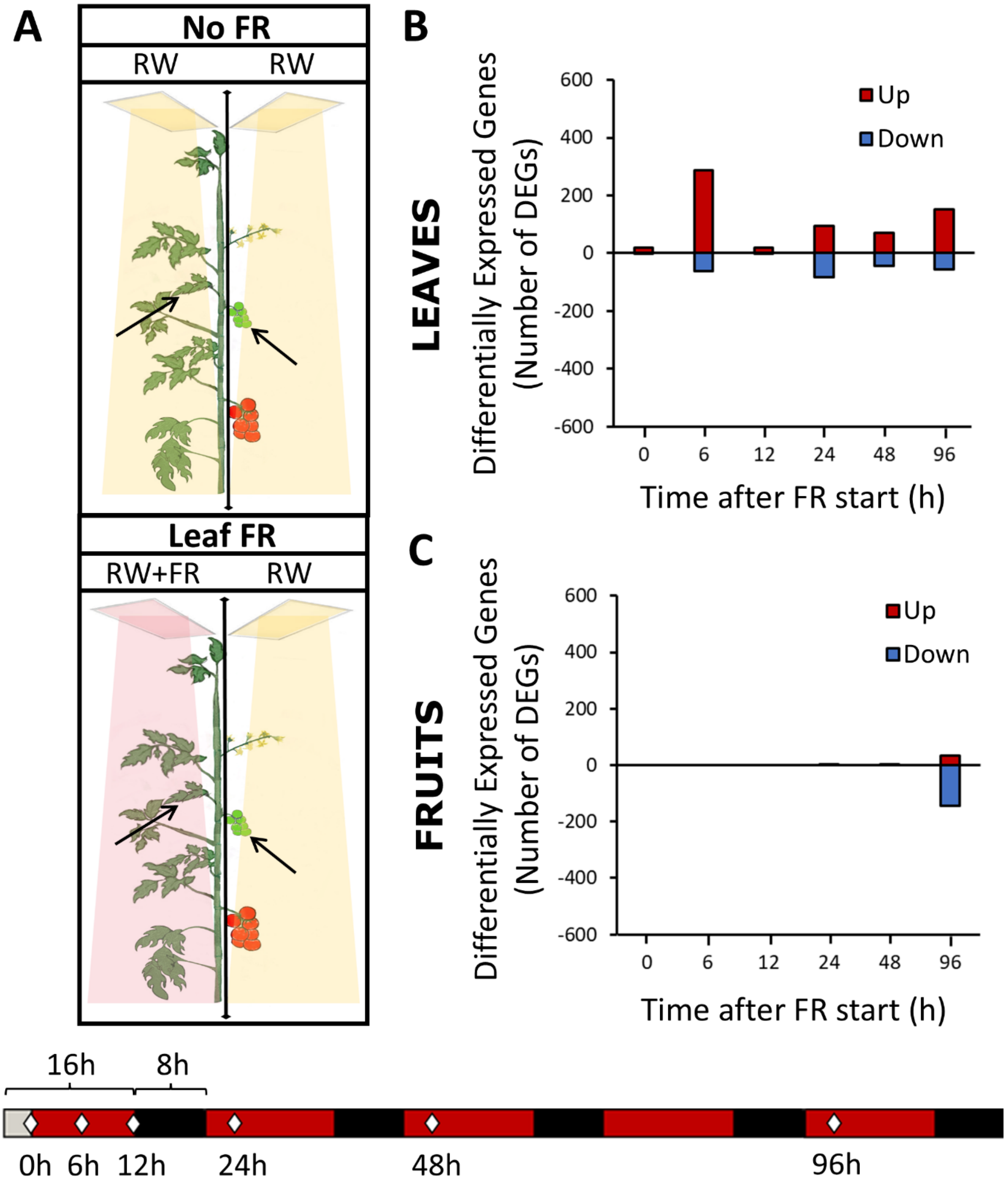

Supplementary Figure S4. Leaf and fruit transcriptomic responses to far-red light (FR) application to vegetative organs (Leaf FR). (A) Plants under Leaf FR or No FR were grown under the same red and white (RW) light until the start of the FR treatment at 63 days after sowing. FR started four hours after the beginning of the RW photoperiod (16/8 Day/Night). Leaf and fruit samples were collected at six time points from pre-exposure (0) to 96 h after the start of FR treatment. Differentially expressed genes (DEGs) were identified for each organ and time point by comparing Leaf FR vs. No FR, using an adjusted p-value threshold of 0.05. DEGs with $Log_2FC > 1$ or < -1 are shown for (B) leaves and (C) fruits.

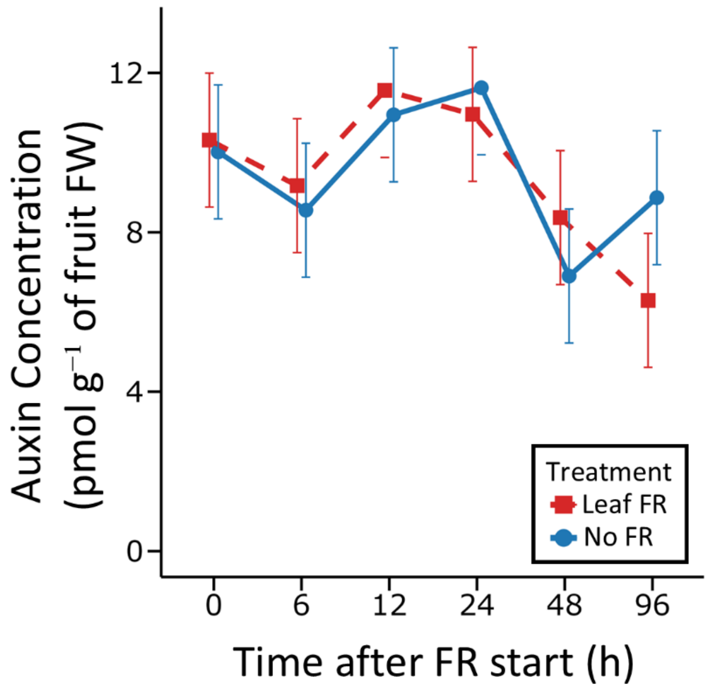

Supplementary Figure S5. Auxin (indole-3-acetic acid) concentration in fruit samples, per time point. Auxin concentration values were analysed with a two-way analysis of variance (ANOVA). Values represent the average of 4 replicates ± SEM (n = 4). Light treatments included application of additional FR to the vegetative organs (Leaf FR) or No FR applied to any organ of the plant.

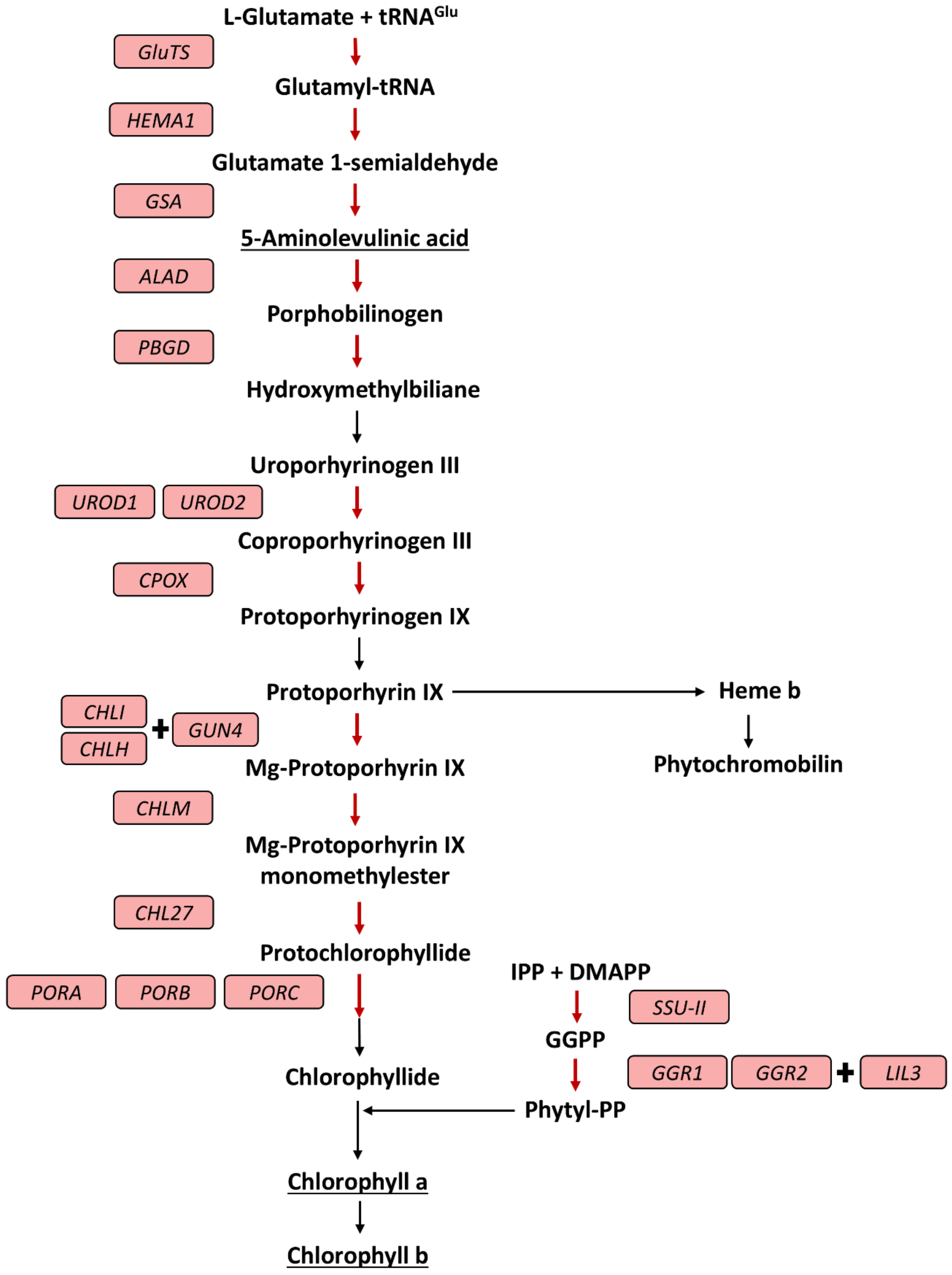

Supplementary Figure S6: Transcriptomic upregulation of chlorophyll biosynthetic genes in fruit samples following far-red light (FR) application to vegetative organs (Leaf FR). Chlorophyll biosynthetic pathway adapted from Kobayashi and Masuda (2016) and Rodriguez-Concepcion et al. (2025). Genes upregulated in fruits 96 h after the start of Leaf FR treatment (adjusted p-value threshold of 0.05) are shown in a red box, with the corresponding metabolic steps indicated by red arrows. Black arrows indicate metabolic steps that are not differentially regulated by the Leaf FR treatment. Abbreviations: GluTS, Glutamyl-tRNA synthase; HEMA, Glutamyl-tRNA reductase; GSA, Glutamate-1-semialdehyde

aminotransferase; ALAD, 5-aminolevulinic acid dehydratase; PBGD, Porphobilinogen deaminase; UROD, Uroporphyrinogen III decarboxylase; CPOX, Coproporphyrinogen III oxidase; CHLI/H, Mg-chelatase ChlI/H; CHLM, SAM Mg-protoporphyrin IX methyltransferase; CHL27, Mg-protoporphyrin IX monomethylester cyclase membrane-bound subunit; POR, Protochlorophyllide oxidoreductase; IPP, isopentenyl diphosphate; DMAPP, dimethylallyl diphosphate; SSU-II, geranylgeranyl diphosphate synthase-like small subunit type 2; GGPP, geranylgeranyl diphosphate; GGR, geranylgeranyl reductase; LIL3, LIGHT-HARVESTING-LIKE 3.

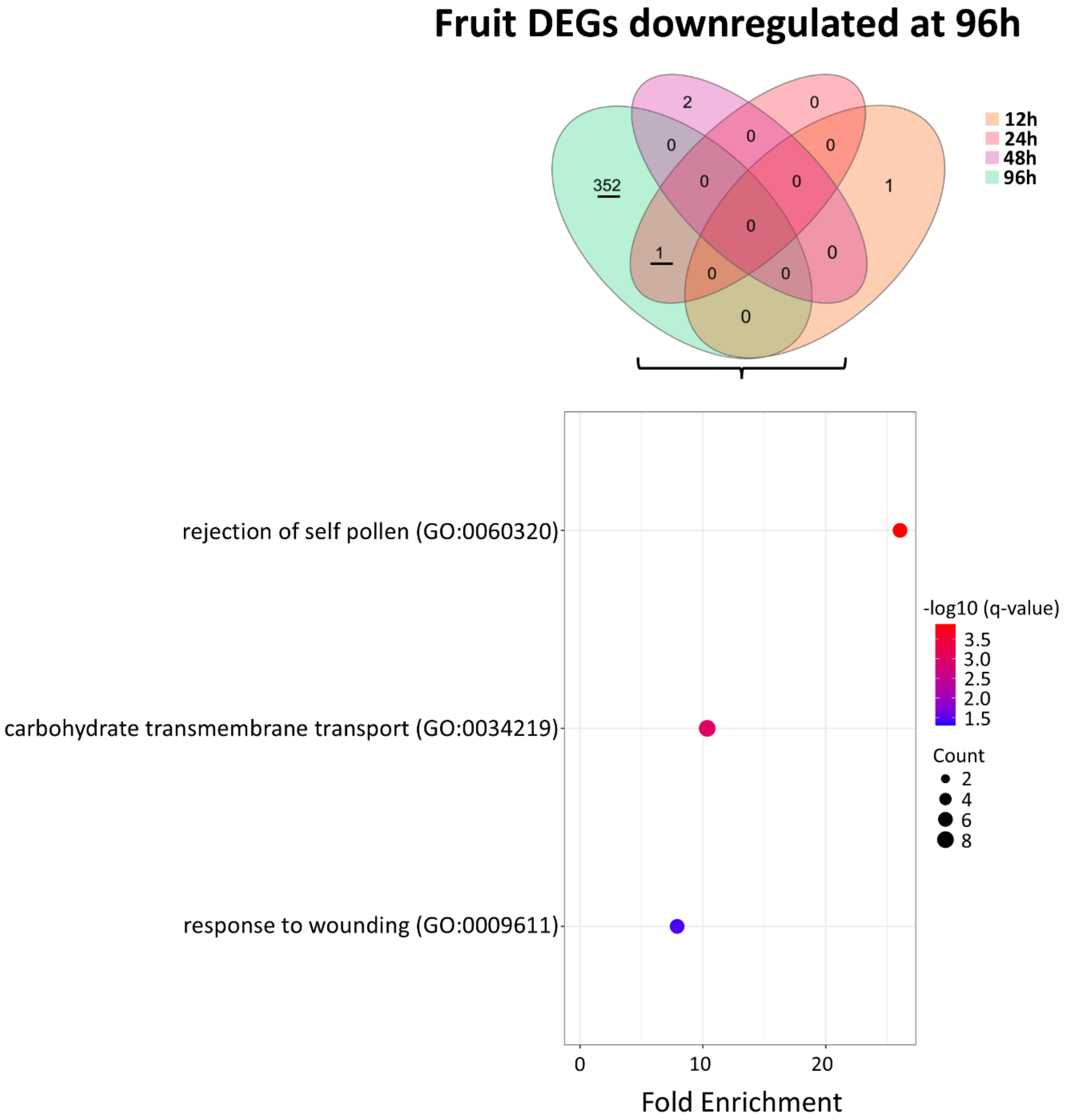

Supplementary Figure S7. Transcriptomic downregulation in fruit samples following far-red light (FR) application to vegetative organs (Leaf FR). Venn diagram of downregulated differentially expressed genes (DEGs) in the fruit samples at different time points, DEGs were identified by comparing Leaf FR vs. No FR treatments, using an adjusted p-value threshold of 0.05. Gene Ontology (GO) categories significantly enriched (adjusted p-value threshold of 0.05) among fruit DEGs downregulated at the last time point (96 h after the FR start).

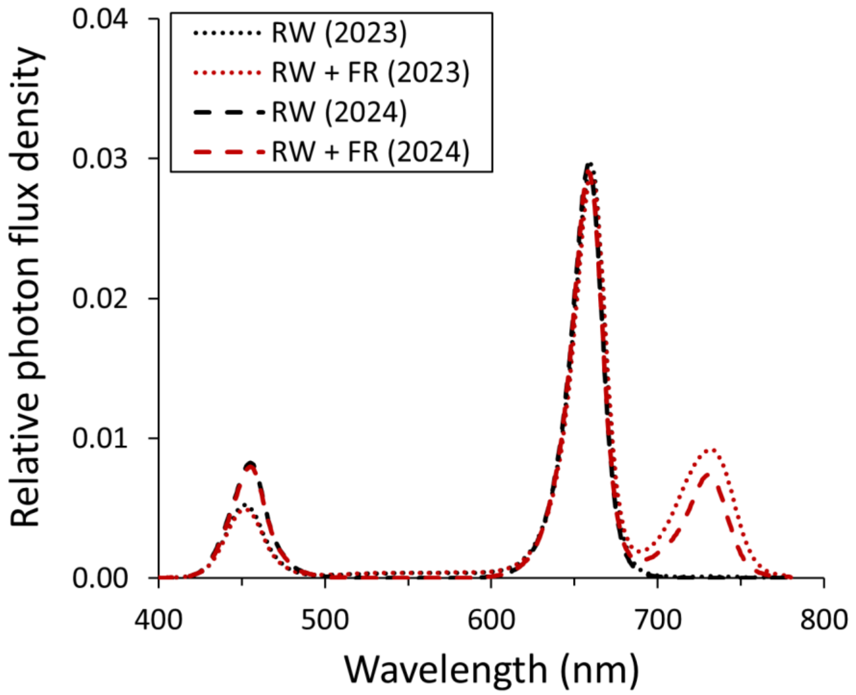

Supplementary Figure S8. Spectral composition of the red and white (RW) and RW + far-red (RW+FR) lighting used in the climate chamber experiments in 2023 and 2024. The light spectra were measured 30 cm below the lamps, before transplant, with a spectrometer (Li-180, Li-Cor Biosciences, Lincoln, NE, USA).

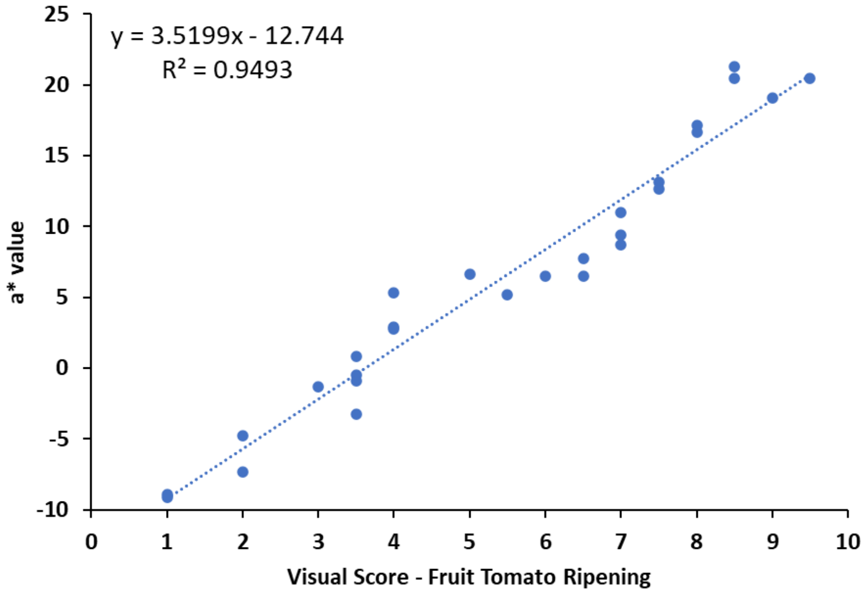

Supplementary Figure S9. Correlation between visual assessment of tomato fruit ripening and colorimeter a* value. Colour-chart standard (The Greenery, Breda, the Netherlands), colorimeter (Minolta CR-400).

Supplementary Tables

Supplementary Table S1: List of abbreviations from Figure 6. Time point is indicated in hours after the start of the far-red light (FR) treatment. In the differentially expressed genes (DEG) column, it is indicated whether the genes were upregulated or downregulated following the FR treatment (adjusted p-value threshold of 0.05).

Time Point	DEG	Gene ID	Gene Abbreviation	Gene Full Name
24, 96	Upregulated	Solyc01g109390	*SlDIR1*	DEFECTIVE IN INDUCED RESISTANCE 1
24, 96	Upregulated	Solyc02g088100	*EXPA5*	Expansin precursor 5
24	Upregulated	Solyc06g065630	*ToFZY*	YUCCA-like flavin monooxygenase
24, 96	Upregulated	Solyc09g090180	*TMF*	Terminating flowering
48	Upregulated	Solyc02g089420	*SlHDG7*	Homeodomain glabrous 7
48	Upregulated	Solyc02g094860	*SlCYP735A1*	Cytochrome P450 - Cytokinins biosynthetic gene
48	Upregulated	Solyc03g006880	*GA20OX1*	Gibberellin-20-oxidase 1
48	Upregulated	Solyc03g006880	*GA2OX1*	Gibberellin-2-oxidase 1
48	Upregulated	Solyc04g077990	*SlLBD38*	Lateral organ boundaries domain 38
96	Downregulated	Solyc01g068410	*SlPIN5*	PIN-formed 5
96	Upregulated	Solyc01g111170	*SlCYP19*	Cyclophillin19
96	Upregulated	Solyc03g120380	*SlIAA19*	Auxin/Indole Acetic Acid 19
96	Upregulated	Solyc03g120450	*PAD-1*	Parental Advice-1 - Aminotransferase
96	Downregulated	Solyc03g121270	*SlIAR3*	IAA-Ala Resistant 3
96	Downregulated	Solyc04g080820	*CKX4*	Cytokinins oxidase 4
96	Upregulated	Solyc05g008060	*SlPIN4*	PIN-formed 4
96	Downregulated	Solyc07g061730	*GA2OX5*	Gibberellin-2-oxidase 5
96	Downregulated	Solyc08g061820	*SlSLOMO*	Arabidopsis thaliana SLOW MOTION
96	Downregulated	Solyc08g061920	*CKX*	Cytokinins oxidase
96	Upregulated	Solyc10g086170	*SlAMI1*	Amidase 1 - indole-3-acetamide (IAM) pathway
96	Downregulated	Solyc11g069500	*SlARF10A*	Auxin response factor 10A
96	Downregulated	Solyc12g042070	*SlARF2*	Auxin response factor 11

Supplementary Table S2: List of abbreviations from Figure 7. The genes were upregulated in the fruit samples by far-red light (FR) application to the vegetative organs (adjusted p-value threshold of 0.05). Time point is indicated in hours after the start of the FR treatment.

Time Point	DEG	Gene ID	Gene Abbreviation	Gene Full Name
96	Upregulated	Solyc04g011350	*2-OGDH*	2-oxoglutarate dehydrogenase
96	Upregulated	Solyc01g109790	*AGPase L1*	Glucose-1-phosphate adenylyltransferase Long subunit 1
96	Upregulated	Solyc07g056140	*AGPase S1*	Glucose-1-phosphate adenylyltransferase Short subunit 1
96	Upregulated	Solyc09g009020	*ER28*	Enolase
96	Upregulated	Solyc04g009030	*GAPDH*	Glyceraldehyde-3-phosphate dehydrogenase
96	Upregulated	Solyc04g082630	*GAPDH*	Glyceraldehyde-3-phosphate dehydrogenase
96	Upregulated	Solyc06g071920	*GAPDH*	Glyceraldehyde-3-phosphate dehydrogenase
96	Upregulated	Solyc05g014470	*GAPDH*	Glyceraldehyde-3-phosphate dehydrogenase
96	Upregulated	Solyc08g079080	*LIN9*	Lycopersicon esculentum invertase 9
96	Upregulated	Solyc04g082880	*PFP subunit α*	Pyrophosphate--fructose 6-phosphate 1-phosphotransferase subunit alpha
96	Upregulated	Solyc02g081160	*PFP subunit β*	Pyrophosphate--fructose 6-phosphate 1-phosphotransferase subunit beta
96	Upregulated	Solyc01g007910	*SCOA1*	Succinate--CoA ligase subunit alpha 1
96	Upregulated	Solyc06g083790	*SCOB*	Succinate--CoA ligase subunit beta
96	Upregulated	Solyc02g091880	*SlCOX6A*	Cytochrome c oxidase subunit 6a
96	Upregulated	Solyc04g074550	*SlCOX6B*	Cytochrome c oxidase subunit 6b
96	Upregulated	Solyc02g062340	*SlFBA2*	Fructose-bisphosphate aldolase 2
96	Upregulated	Solyc09g075450	*SlFUM1*	fumarase 1
96	Upregulated	Solyc08g083320	*SlGBSS1*	Starch synthase
96	Upregulated	Solyc03g121070	*SlHXK1*	Hexokinase 1
96	Upregulated	Solyc03g115990	*SlMDH*	malate dehydrogenase
96	Upregulated	Solyc08g066100	*SlPFK3*	ATP-dependent 6-phosphofructokinase 3
96	Upregulated	Solyc07g066600	*SlPGK*	Phosphoglycerate kinase
96	Upregulated	Solyc03g083090	*SlSS1*	Starch synthase 1
96	Upregulated	Solyc04g011510	*SlTPI*	Triosephosphate isomerase
96	Upregulated	Solyc02g093680	*SUCDH*	Succinate dehydrogenase
96	Upregulated	Solyc12g009300	*SUS1*	Sucrose synthase 1
96	Upregulated	Solyc07g042550	*SUS3*	Sucrose synthase 3

Supplementary Table S3: Mineral composition of standard nutrient solution for tomato fertigation.

Mineral composition	Dose
NH_4^+	1.2 mM
K^+	11.0 mM
Ca^{2+}	6.3 mM
Mg^{2+}	2.8 mM
NO_3^-	18.4 mM
SO_4^{2-}	5.1 mM
PO_4^{2-}	1.7 µM
Fe^{3+}	25.0 µM
Mn^{2+}	10.0 µM
Zn^{2+}	5.0 µM
$H_2BO_3^-$	30.0 µM
Cu^{2+}	0.8 µM
MoO_4^{2-}	0.5 µM

Supplementary Methodology

Supplementary method S1. Carbohydrate extraction and quantification

15 mg of freeze-dried fruit tissue is weighed into a 12 mL centrifuge tube, and the precise weight is recorded. Ethanol extraction is performed by adding 5 mL of 80% ethanol to each sample. The tubes are vortexed before being incubated in a shaking water bath at 80°C for 20 minutes. After incubation, the samples are vortexed again.

Centrifugation follows at 8,500 rcf and 4°C for 5 minutes. 1 mL of the resulting supernatant is then transferred to 1.5 mL Eppendorf tubes for soluble sugars quantification, while the centrifuge tube with the remaining sample will be used for starch quantification.

Soluble sugars extraction:

The supernatant in the Eppendorf tube is dried using a Savant SpeedVac rotary evaporator for 2 hours (SPD2010, Thermo Fisher Scientific, Waltham, MA, USA). At the end of the drying step, 1 mL of MilliQ water is added to each sample, followed by vortexing. The samples undergo 10 minutes of sonication before being centrifuged at maximum speed and 4°C for 10 minutes. Samples were diluted with MilliQ water at a 20x ratio for immature green fruits in Expt. 2 and a 50x ratio for ripe fruits in Expt. 1, and sucrose, fructose, and glucose are quantified.

Starch extraction:

The remaining supernatant present in the centrifuge tube is discarded, while the pellet undergoes a wash step.
Wash step: 3 ml of ethanol 80% are added to the pellet in the centrifuge tube, mixed well by vortexing, and then centrifuged at 8,500 rcf and 4°C for 5 min. Supernatant is discarded, and the wash step is repeated for a total of 3 times.

The pellet is dried using a Savant SpeedVac rotary evaporator for at least 20 minutes.

Two empty tubes are added to the sample tubes for blank determination. 2 mL of alpha-amylase solution (1 mg/mL alpha-amylase Rohalase in MilliQ water) is added to each tube. The tubes are vortexed before being incubated in a shaking water bath at 90°C for 30 minutes.

1 mL of amyloglucosidase (0.5 mg/mL in 50 mM citrate buffer, pH = 4.6) is added to each tube. The tubes are vortexed before being incubated in a shaking water bath at 60°C for 10 minutes.

Centrifugation follows at 8,800 rcf and ambient temperature for 5 minutes. 1 mL of the resulting supernatant is then transferred to 1.5 mL Eppendorf tubes and centrifuged at maximum speed and ambient temperature for 15 minutes. Before analysis, samples are diluted with MilliQ water at a 50x ratio, and degraded starch is quantified as glucose.

Carbohydrate quantification:

Carbohydrate quantification is carried out using a high-performance ion chromatograph (ICS-5000, Thermo Fisher Scientific) equipped with an anion exchange CarboPac PA1 column (250 mm) at 25°C. The eluent consists of 100 nM NaOH at a flow rate of 0.25 mL/min. Detection is performed using pulsed amperometry, and chromatogram analysis, along with sugar concentration quantification, is conducted using Chromeleon software (Thermo Fisher Scientific).

Supplementary method S2. Pipeline for analysis of RNA sequencing data

Raw reads were filtered using SOAPnuke (software version 1.5.6, GitHub - SOAPnuke), removing low-quality reads, reads containing adapters, and reads with unknown base content greater than 0.1%. Clean reads were aligned to the reference tomato genome ITAG4. 0 using Bowtie2 (software version 2.5.0, Bowtie 2). Gene expression quantification was carried out using RSEM (software version 1.3.1, GitHub - RSEM), and differentially expressed genes were identified using DESeq2 (software version 1.40.2, Bioconductor - DESeq2) and an adjusted p-value (Benjamini-Hochberg q-value) threshold of 0.05. Gene Ontology (GO) enrichment analysis of the differentially expressed gene set was carried out using Fisher's exact test implemented in PANTHER (version 19.0, PANTHER) and an adjusted p-value (Benjamini-Hochberg q-value) threshold of 0.05.
